# Acyl-CoA synthetase 6 controls rod photoreceptor function and survival by shaping the phospholipid composition of retinal membranes

**DOI:** 10.1038/s42003-024-06691-8

**Published:** 2024-08-21

**Authors:** Yixiao Wang, Silke Becker, Stella Finkelstein, Frank M. Dyka, Haitao Liu, Mark Eminhizer, Ying Hao, Richard S. Brush, William J. Spencer, Vadim Y. Arshavsky, John D. Ash, Jianhai Du, Martin-Paul Agbaga, Frans Vinberg, Jessica M. Ellis, Ekaterina S. Lobanova

**Affiliations:** 1 Department of Ophthalmology, University of Florida, Gainesville, FL USA; 2 Department of Ophthalmology, University of Utah, Salt Lake City, UT USA; 3 Department of Ophthalmology, Duke University, Durham, NC USA; 4 Department of Ophthalmology, University of Pittsburgh, Pittsburgh, PA USA; 5 Departments of Ophthalmology and Visual Sciences and Biochemistry and Molecular Medicine, West Virginia University, Morgantown, WV USA; 6 Department of Ophthalmology, University of Oklahoma Health Sciences Center and Dean McGee Eye Institute, Oklahoma City, OK USA; 7 Department of Ophthalmology and Visual Sciences, SUNY Upstate Medical University, Syracuse, NY USA; 8 East Carolina University, Greenville, NC USA

**Keywords:** Cell death in the nervous system, Mechanisms of disease

## Abstract

The retina is light-sensitive neuronal tissue in the back of the eye. The phospholipid composition of the retina is unique and highly enriched in polyunsaturated fatty acids, including docosahexaenoic fatty acid (DHA). While it is generally accepted that a high DHA content is important for vision, surprisingly little is known about the mechanisms of DHA enrichment in the retina. Furthermore, the biological processes controlled by DHA in the eye remain poorly defined as well. Here, we combined genetic manipulations with lipidomic analysis in mice to demonstrate that acyl-CoA synthetase 6 (*Acsl6*) serves as a regulator of the unique composition of retinal membranes. Inactivation of *Acsl6* reduced the levels of DHA-containing phospholipids, led to progressive loss of light-sensitive rod photoreceptor neurons, attenuated the light responses of these cells, and evoked distinct transcriptional response in the retina involving the Srebf1/2 (sterol regulatory element binding transcription factors 1/2) pathway. This study identifies one of the major enzymes responsible for DHA enrichment in the retinal membranes and introduces a model allowing an evaluation of rod functioning and pathology caused by impaired DHA incorporation/retention in the retina.

## Abstract (web summary)

Acyl-CoA synthetase 6 (Acsl6)-facilitated enrichment of docosahexaenoic acid (DHA) in the retina is essential for rod photoreceptor function, viability and control of basal activity of the Srebf1/2 pathway.

## Introduction

Retinal phospholipids have a complex fatty acid composition that is not found in other tissues, with a high content of polyunsaturated fatty acids (PUFAs) and significant enrichment with omega-3 docosahexaenoic fatty acid (DHA)^1–4^. DHA content is particularly high in rod outer segment phospholipids, accounting for at least 50% of the total fatty acid pool^1–3^. The mechanisms responsible for maintaining the high levels of polyunsaturated fatty acids in the retina remain poorly understood. Dietary and labeling studies have demonstrated that DHA is massively acquired in the retina and brain during development^5,6^. After development is complete, brain and retina rely upon a continuous but minor supply of DHA from blood, and DHA retention and recycling behind the blood-retina barrier (BRB) through undefined mechanisms.

For many years, it was erroneously believed that DHA accumulation in neuronal tissues did not require specific transporters and that DHA crosses the blood-brain barrier (BBB) and BRB in non-esterified (NE-DHA) form by diffusion^5,7^. In this model of transport, DHA dissociates from albumin in the blood, diffuses across the cell membranes that form the blood–organ barriers, and then moves between cells and cellular compartments while bound to fatty acid-binding proteins. Surprisingly, several years ago, it was discovered that DHA accretion by the brain and retina relies heavily upon a lysolipid transporter called major facilitator superfamily domain-containing 2A (Mfsd2a)^8–10^. This transporter is expressed in the cells that form the blood-brain and blood-retina barriers and transports phospholipids with a single fatty acid (lysophospholipids) rather than the standard two-fatty acid-containing form^8–13^. Genetic deletion of *Mfsd2a* in mice leads to a twofold depletion of DHA in the retina and brain without systemic changes^8,9^. These studies provided evidence that DHA delivery in the lysophospholipid form is a major route of its transport across the blood-retina barrier (BRB) but highlighted the intriguing questions regarding the mechanisms responsible for DHA retention in the retina.

Alterations in retinal phospholipids and fatty acid profiles, as well as omega-3 depletion, have been observed in many inherited and age-related retinal diseases, as well as in diabetic retinopathy, and these alterations have been proposed to be contributing factors driving pathology and disrupting photoreceptor light responses^14–23^. Previous attempts to understand the roles of polyunsaturated fatty acids in photoreceptors relied on dietary manipulations in laboratory models that involved feeding omega-3-deficient diets for generations. However, the interpretations of these studies were complicated due to substitutions of naturally present DHA with structurally similar omega-6 fatty acids that are generally not found in the retina^24,25^. Therefore, the identification of molecular mechanisms responsible for the unique composition of retinal phospholipids and the development of genetic models of retina-specific DHA deficiency would circumvent the remaining barriers in determining the biological processes controlled by polyunsaturated fatty acids in the retina.

Depletion of DHA in the retina was previously reported in several mouse models. One group proposed roles for the *Adipor1* (Adiponectin receptor 1) and *Mfrp* (Membrane frizzled-related protein) genes in DHA retention in photoreceptors^26,27^. Note, however, that the specificity of protein products of these genes for DHA or DHA-containing lipids has yet to be demonstrated. Adipor1 is a ubiquitously expressed gene thought to be involved in regulating AMPK metabolism or to function as a ceramidase^28,29^. *Mfrp* is a gene of unknown function highly enriched in the retinal pigment epithelium (RPE)^30^. Additionally, DHA depletion in these models could take place secondary to the ongoing stress (e.g., dysregulation of AMP-activated protein kinase signaling in *Adipor1*^*−/−*^ mice, or RPE defects impacting outer segment phagocytosis and lipid recycling in *Mfrp* mutant mice), photoreceptor loss^28^ and/or shortening of photoreceptor outer segments containing particularly high level of DHA^2,30^. More recently, it was reported that mutation in the *Tmem135* (transmembrane protein 135) gene and genetic inactivation of *Lpaat3* (lysophosphatidic acid acyltransferase 3) impacted DHA levels in multiple tissues, including the liver and the retina^31–33^. *Tmem135* is a multifunctional protein proposed to be involved in peroxisomal DHA metabolism and mitochondrial dynamics^33–35^. Lysophosphatidic acid acyltransferases are the principal enzymes responsible for phospholipid synthesis in every cell^36^. An attribution of retinal phenotype in *Tmem135* mutant and *Lpaat3*^−/−^ mice exclusively to DHA depletion is complicated by systemic changes in lipid metabolism, the pleiotropic nature of *Tmem135*, and the potential impact on overall phospholipid synthesis in the retinas of *Lpaat3*^−/−^ mice.

Acyl-CoA long-chain synthetases are enzymes responsible for the conversion of long-chain free fatty acids to Acyl-CoAs before their incorporation into membrane phospholipids or β-oxidation^37^. A high-affinity interaction of Acyl-CoA synthetase 6 (*Acsl6*) with DHA is well-documented^38^. More recent studies showed a major role of Acsl6 in DHA enrichment in the neurons of the brain in the absence of systemic lipid changes^39,40^. We now report that genetic inactivation of *Acsl6* reduces the levels of DHA-containing phospholipids in the retina, drives the accumulation of inflammatory cells in the subretinal space, profoundly impacts rod photoreceptor light responses, leads to their progressive loss with age, and evokes a distinct lipogenic transcriptional response. An analysis of retina-specific *Acsl6* knockout mice demonstrated the role of the retinal Acsl6 pool in DHA enrichment/ retention behind the blood-retina barrier. The study identifies one of the key enzymes responsible for DHA enrichment in the retina and introduces a genetic model that is likely to facilitate the characterization of retinal pathology caused by impaired DHA incorporation into retinal phospholipids. The findings highlight an altered fatty acid profile of phospholipids as a potent driver of photoreceptor dysfunction and degeneration, calling to re-evaluate the pathological mechanisms in all forms of retinal degeneration that impact, directly or indirectly, DHA retention/delivery to the retina.

## Results

### *Acsl6* transcripts are found in all retinal neurons, Muller Glia, and Retinal Pigment Epithelium

**Fig. 1:**
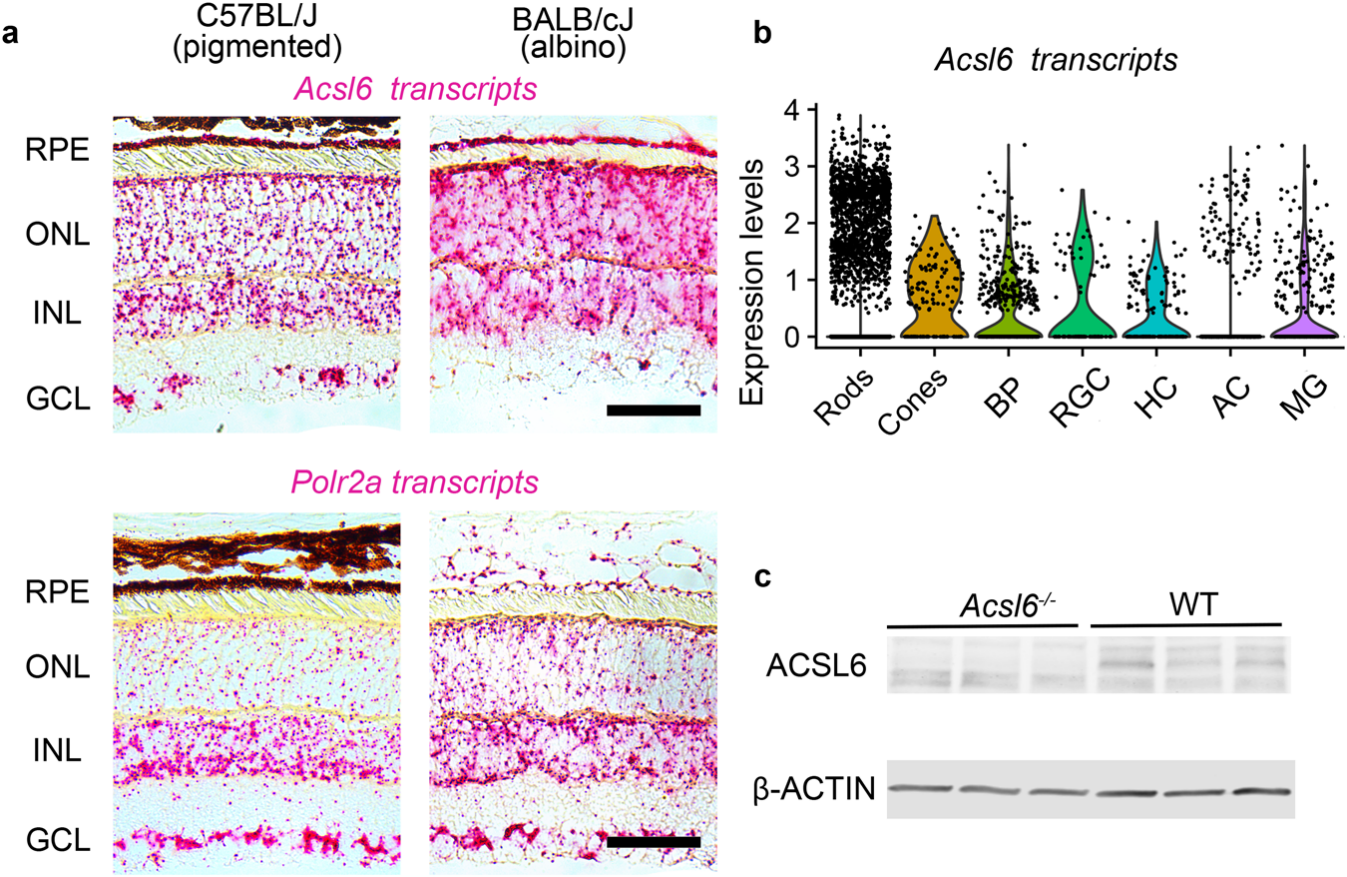
*Acsl6* expression in the retina. **a** *Acsl6* transcripts detected in the retinas of pigmented C57BL/J and albino BALB/CJ wild-type mice via RNA in situ hybridization (ISH). A probe for *Polr2a* (DNA-directed RNA polymerase II subunit RPB1) was used as a positive control to assess tissue quality. ONL outer nuclear layer (containing photoreceptor nuclei), INL inner nuclear layer, GCL ganglion cell layer, RPE retinal pigment epithelium. Scale bar, 50 μm. See also Supplementary Fig. 1 for cross-sections of entire retinas. **b** *Acsl6* transcripts detected in indicated cell types in the scRNA-seq dataset prepared from wild-type mouse retinas. BP bipolar cells, RGC retinal ganglion cells, HC horizontal cells, AC amacrine cells, MG Muller glia. **c** Western blots of ACSL6 and β-ACTIN proteins in retinas of one-month-old *Acsl6*^*−/−*^ mice and their WT littermates.

In our first set of experiments, we investigated *Acsl6* expression in the mouse retina. RNA in situ hybridization (RNA ISH) analysis showed pan-retinal staining for *Acsl6* transcripts in all retinal layers (Fig. 1a, see also Supplementary Fig. 1), including the outer nuclear layer (ONL), which contains photoreceptor nuclei, the inner nuclear layer (INL), and the ganglion cell layer (GCL). Prominent staining for *Acsl6* transcripts was also detected in the retinal pigment epithelium layer and was particularly evident from the analysis of retinal sections prepared from albino (BALB/cJ) mice, in which chromogenic stain is not masked by pigment. Single-cell transcriptomic datasets prepared from wild-type mouse retinas identified *Acsl6* transcripts in all retinal neurons and Muller glia (Fig. 1b). To test the role of Acsl6 in the regulation of phospholipid composition in the retina, we used previously developed whole-body *Acsl6* knockout mice (*Acsl6*^*−/−*^)^39^. Western blot analysis confirmed a complete loss of ACSL6 protein (Fig. 1c) in the retinas of *Acsl6*^*−/−*^ mice.

### Progressive rod photoreceptor loss occurs in *Acsl6*^−/−^ mice

**Fig. 2:**
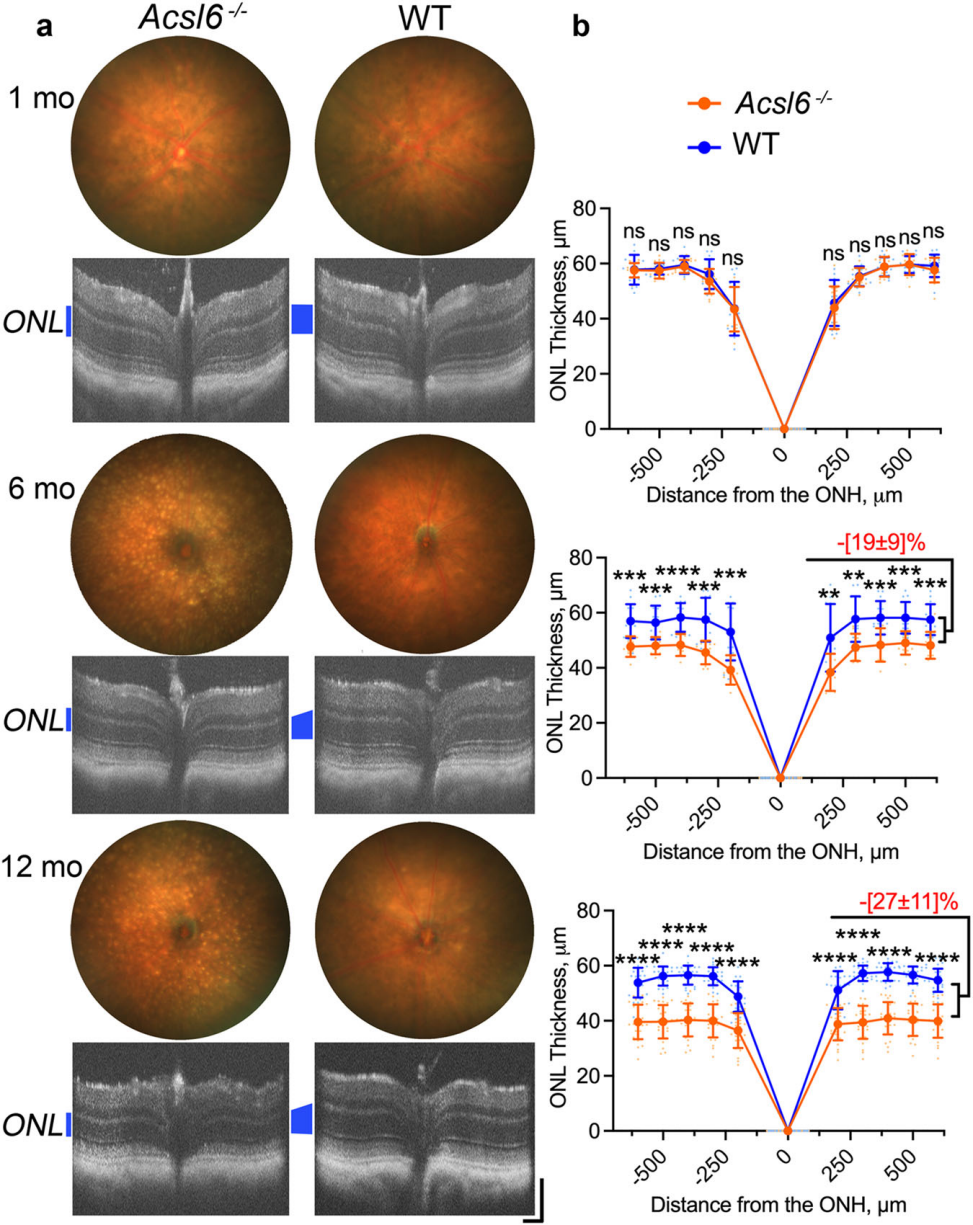
OCT and fundoscopy analysis of *Acsl6*^*−/−*^ mice. **a** Representative funduscopic images and horizontal SD-OCT  (Spectral Domain-Optical Coherence Tomography) scans from *Acsl6*^*−/−*^ and WT littermate mice at different ages (temporal-nasal from left to right). **b** Horizontal OCT spider diagrams of the outer nuclear layer (ONL) thickness for mice of indicated genotypes at 1, 6 and 12 months of age. The ONL is marked with a blue vertical line on OCT images; scale bar, 100 μm. To estimate ONL thickness changes, measurements from OCT spider diagrams for each mouse were summed and expressed as a percentage of the average value calculated for WT littermates. The data are presented as mean ± SD. The number of eyes analyzed was as follows: 1 mo, *Acsl6*^*−/−*^—13 and WT—13, 6 mo, *Acsl6*^*−/−*^—11 and WT—14, 12 mo, *Acsl6*^*−/−*^—26 and WT—24. Quantification was performed by individuals not aware of specific genotypes.

**Fig. 3:**
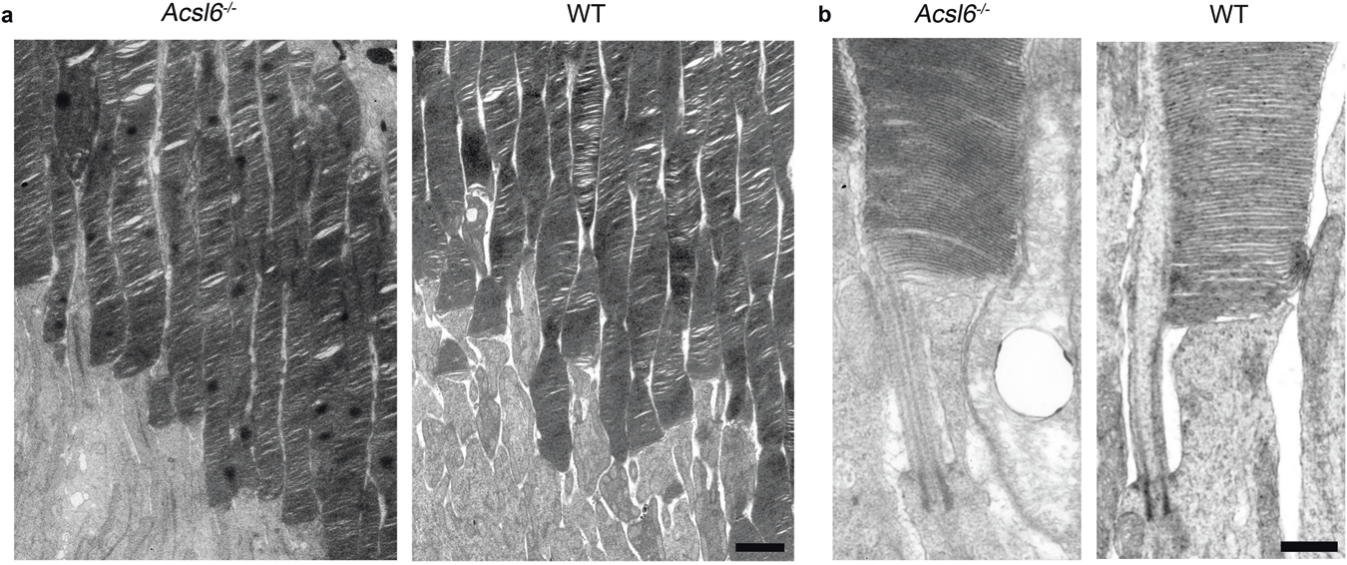
Ultrastructural analysis of rod photoreceptor outer segments in *Acsl6*^*−/−*^ mice. **a** Representative transmission electron microscopy images rod of outer segment areas from *Acsl6*^*−/−*^ and WT mice and **b** the inner-outer segment junction regions of individual photoreceptors. Scale bars: 2 μm in **a** and 0.5 μm in **b**.

To select critical time points for analysis, we first probed morphological changes in Acsl6 knockout mice at different ages using two in vivo imaging methods, optical coherence tomography (OCT) and fundoscopy. As shown in Fig. 2, at one month of age, the structure of the retina appeared normal in *Acsl6*^*−/−*^ mouse line and was indistinguishable from that of their wild-type littermates. Longitudinal studies over one year detected slow thinning of the ONL, indicating photoreceptor loss which was reliably detected in animals past six months of age. Consistent with the OCT analysis, retinal cross-sections prepared from young 2-month-old *Acsl6*^*−/−*^ showed normal structure (Supplementary Fig. 2a, b and Fig. 3a) and were indistinguishable from those of their wild-type littermates. A substantial loss of the nuclei (at least 30%) in the outer retina containing mostly rod photoreceptor nuclei was evident in the older (12-month-old) animals (Supplementary Fig. 2c, d and Fig. 3b).

Fundus images of young (one-month-old) *Acsl6* knockout mice appeared normal (Fig. 2a), but we consistently detected multiple hyperreflective dots in mice past 3 months of age. The number and size of hyperreflective dots continued to increase with age. Previous studies ascribed similar fundus objects in other animal models to an ongoing inflammatory response and the accumulation of microglia or monocyte-derived recruits in the subretinal space, which have been observed in several models of photoreceptor degeneration^41–43^. These cells could be detected with a commonly used microglia/macrophage-specific marker, Iba1 (Ionized calcium-binding adapter molecule 1). As predicted, confocal images of retinal flat mounts of outer segment areas showed many Iba1-positive cells (Supplementary Fig. 4) in older (12-month-old) *Acsl6*^*−/−*^ mice. Only scarce Iba1 staining was detected in younger (one-month-old) *Acsl6*^*−/−*^ animals, whose fundus images appeared normal.

A modified PUFA profile could potentially destabilize outer segment formation and maintenance in photoreceptors by altering the physical properties of membranes^1–3,44^. Therefore, in the next set of experiments we used electron microscopy to study changes in the outer retina structure of *Acsl6*^*−/−*^ mice. Since retinal degeneration in these mice was slow, we limited our analysis to older 9-month-old mice in which photoreceptor loss was evident. The ultrastructural analysis was limited to the rods, major cells in the retina accounting for 97% of photoreceptors in the mouse retina. As shown in Fig. 3, the photoreceptors at this age had normally looking outer segments and were indistinguishable from wild-type mice. Consistent with ongoing retinal degeneration, we occasionally observed rods with ruptured or distorted outer segments, immune cells in the outer segment area, vesicular material in the extracellular space, and swollen mitochondria in the photoreceptor soma (Supplementary Fig. 5). Considering that the majority of rod photoreceptors retained structurally normal outer segments, it seems unlikely that the loss of *Acsl6* and changes in the phospholipid composition cause prominent ultrastructural damage. Rather, we suggest that occasional pathological defects reflect photoreceptor stress or represent photoreceptor debris at the last stages of their fragmentation and death.

### *Retinas from Acsl6*^*−/−*^ and *Mfsd2a*^*−/−*^ mice display similar changes in the phospholipid profiles

**Fig. 4:**
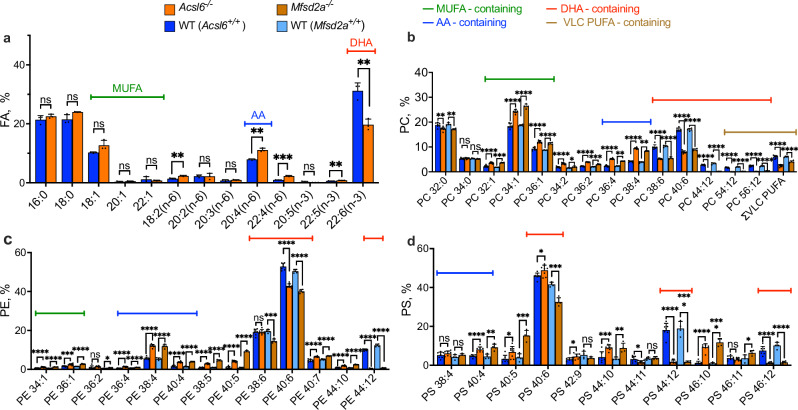
Changes in the fatty acid and phospholipid composition caused by loss of *Acsl6.* **a** Fatty acid profile of the retinas from *Acsl6*^*−/−*^ and WT mice, shown as % of total fatty acid content. *Acsl6*^*−/−*^ — 3 and WT (*Acsl6*^*+/+*^) — 3. Comparison of phospholipid profiles for (**b**) phosphatidylcholine (PC), (**c**) phosphatidylethanolamine (PE) and (**d**) phosphatidylserine (PS) phospholipids are shown as percentages within the corresponding class of indicated mice. The data are presented as mean ± SD. The number of samples used for phospholipid analysis was as follows: *Acsl6*^*−/−*^— 8 and WT (*Acsl6*^*+/+*^) — 7; *Mfsd2a*^*−/−*^ — 4 and WT (*Mfsd2a*^*+/+*^) — 4. MUFA monounsaturated fatty acid, AA arachidonic fatty acid, DHA docosahexaenoic fatty acid, VLC PUFA very-long-chain polyunsaturated fatty acid. All analyzed mice were two-month-old.

Based on the morphological findings (Fig. 2, Supplementary Figs. 2 and 3), we proceeded with lipidomics analysis in 2-month-old animals. At this age, photoreceptors are fully mature in wild-type animals, and no overt abnormalities are detected in the retinas of *Acsl6*^*−/−*^ mice. Consistent with our hypothesis, the fatty acid analysis of the total retinal lipid extracts from *Acsl6*^*−/−*^ mice showed a reduced level of DHA, which was compensated by an increased level of omega-6 fatty acids, including arachidonic acid (Fig. 4a). Next, we compared the uniquely diverse fatty acid profiles of retinal phosphatidylcholines (PC) among animal groups. As expected, *Acsl6*^*−/−*^ mice had reduced levels of DHA-containing phosphatidylcholines (Fig. 4b, red bracket). Additional changes included reduced levels of very-long-chain polyunsaturated fatty acid-containing phospholipids (VLC PUFAs, Fig. 4b, yellow bracket) which are important for human health and are thought to be derived from DHA^18,45^, as well as a compensatory increase in major phospholipids containing arachidonic acid (AA, Fig. 4b, blue bracket) and monounsaturated fatty acids (MUFAs. Figure 4b, green bracket). Similarly, phosphatidylethanolamine (PE) and phosphatidylserine (PS) phospholipids displayed a reduction in DHA-containing species, which was counteracted by an elevation in AA-containing phospholipids (Fig. 4c, d). Intriguingly, the changes in the phospholipid profiles of *Acsl6*^*−/−*^ retinas were nearly identical to those observed in the retinas of *Mfsd2a*^*−/−*^ mice (Fig. 4bc, d)^10^. Notably, phospholipids with DHA in both sn-1 and sn-2 positions of the glycerol backbone (PC 44:12, PE 44:12, PS 44:12), exclusively enriched in the retina^46^, almost entirely disappeared in both mice.

An increasing number of studies have focused on complex adaptational changes in glucose metabolism of the retina and the intimate connection between glucose and fatty acid/lipid metabolism under some pathological conditions^47–49^. However, our analysis of principal metabolites of glycolysis and TCA cycle in *Acsl6*^*−/−*^ retinas indicated that the phospholipid remodeling described above takes place without notable metabolic changes in these pathways (Supplementary Fig. 6).

Next, we generated retina-specific *Acsl6* knockout mice (*Acsl6*^Retina KO^) to determine the contribution of local retinal Acsl6 pool to maintaining DHA levels. This strain was developed by crossing mice bearing the floxed *Acsl6* allele with *Chx10-Cre* mice, which express CRE recombinase in all retinal neurons and Muller glia early in the development^50,51^. The progression of ONL thinning and rod photoreceptor loss in *Acsl6*^*−/−*^ and *Acsl6*^Retina KO^ mice was nearly identical (Supplementary Figs. 7, 2 and 3) and the difference in the progression of photoreceptor loss between the lines was not statistically significant. The change in the acyl profiles of phospholipids in *Acsl6*^*−/−*^ and *Acsl6*^*Retina KO*^ mice were also similar (Supplementary Fig. 8): a depletion of DHA-containing phospholipids that was compensated with elevated levels of MUFA- and AA-containing phospholipids. These findings demonstrate that retinal *Acsl6* is primarily responsible for DHA enrichment behind BRB independent of systemic changes in DHA synthesis, metabolism, and transport. Interestingly, the phospholipids containing di-DHA in sn-1 and sn-2 positions of the glycerol backbone (PC 44:12 and PE 44:12) were lost entirely in the retinas of *Acsl6*^*−/−*^ mice and only partially reduced in *Acsl6*^Retina KO^ mice. This observation might be indicative of the contribution of Acsl6 in other cells, such as RPE, to maintaining high DHA levels behind the BRB. Alternatively, this difference could be explained by incomplete inactivation of the *Acsl6* gene in all retinal cells of *Acsl6*^*Retina KO*^ retinas and it might be caused by known mosaicism of the transgenic *Cre* line. Indeed, the extent of ACSL6 depletion in the *Acsl6*^Retina KO^ retinas analyzed with western blotting varied between animals from complete loss to the presence of trace levels (Supplementary Fig. 9).

Considering the overall similarity in the phospholipid changes and the progression of rod photoreceptor loss, and to avoid challenges with a potentially incomplete loss of *Acsl6* in all retinal cells of *Acsl6*^*Retina KO*^ mice, all further analysis was limited to the whole-body *Acsl6*^*−/−*^ knockout mice.

### Attenuation of photoreceptor light responses precedes age-related retinal degeneration in *Acsl6*^*−/−*^ mice

**Fig. 5:**
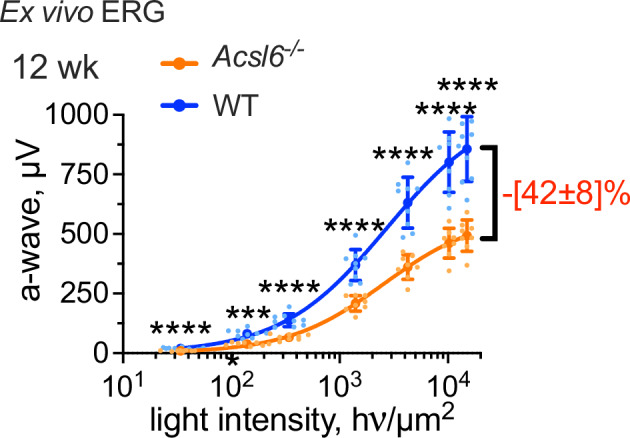
Ex vivo ERG responses in *Acsl6*^*−/−*^ knockout mice. Pharmacologically isolated light responses from rod photoreceptors were measured with ex vivo ERG in the retinas of 12-week-old mice and their littermates in response to light flashes of increasing intensity. Number of retinas: *Acsl6*^*−/−*^ — 8, WT— 9. Reduction in response at maximum light intensity computed as a percentage of the average values for wild-type littermate mice. The data are presented as mean ± SD.

The presence of multiple unsaturated double bonds in PUFA molecules introduces kinks into their structure, which increases membrane disorder and affects the physical properties of membranes. These changes impact the conformation and the functional characteristics of transmembrane and peripheral proteins and, ultimately, signaling characteristics of the pathways to which these proteins belong^52^. The phototransduction is a diffusion-regulated process, therefore, it is generally thought that high PUFA content in photoreceptors may alter the efficiency of phototransduction through modifications of the biophysical properties of membranes^1–4,53–59^.Therefore, in the next set of experiments, we studied whether and how the depletion of PUFA impacts rod function in *Acsl6*^*−/−*^ mice. We again focused on rods and used ex vivo ERG measurements allowing us to isolate rod photoresponses pharmacologically^60^. In this experimental protocol, the responses from bipolar cells are completely suppressed, while cone responses are negligible and fall within the noise of these measurements since cones comprise only 3% of photoreceptors in mice. As shown in Fig. 5, dark-adapted flash ERG responses of rods were substantially decreased (~40% reduction of the maximal a-wave amplitude) in 12-week-old *Acsl6*^*−/−*^ mice, as compared to their wild-type littermates. This reduction indicates a substantial functional defect that could not be attributed to rod photoreceptor loss at this age. As expected, rod photoreceptor function in *Acsl6*^*−/−*^ mice continued declining with age due to ongoing stress and degeneration (Supplementary Fig. 10).

### Elevated activity of *Srebf1/2* pathway as a characteristic transcriptional stress response to the impaired DHA transport/retention in the retina

**Fig. 6:**
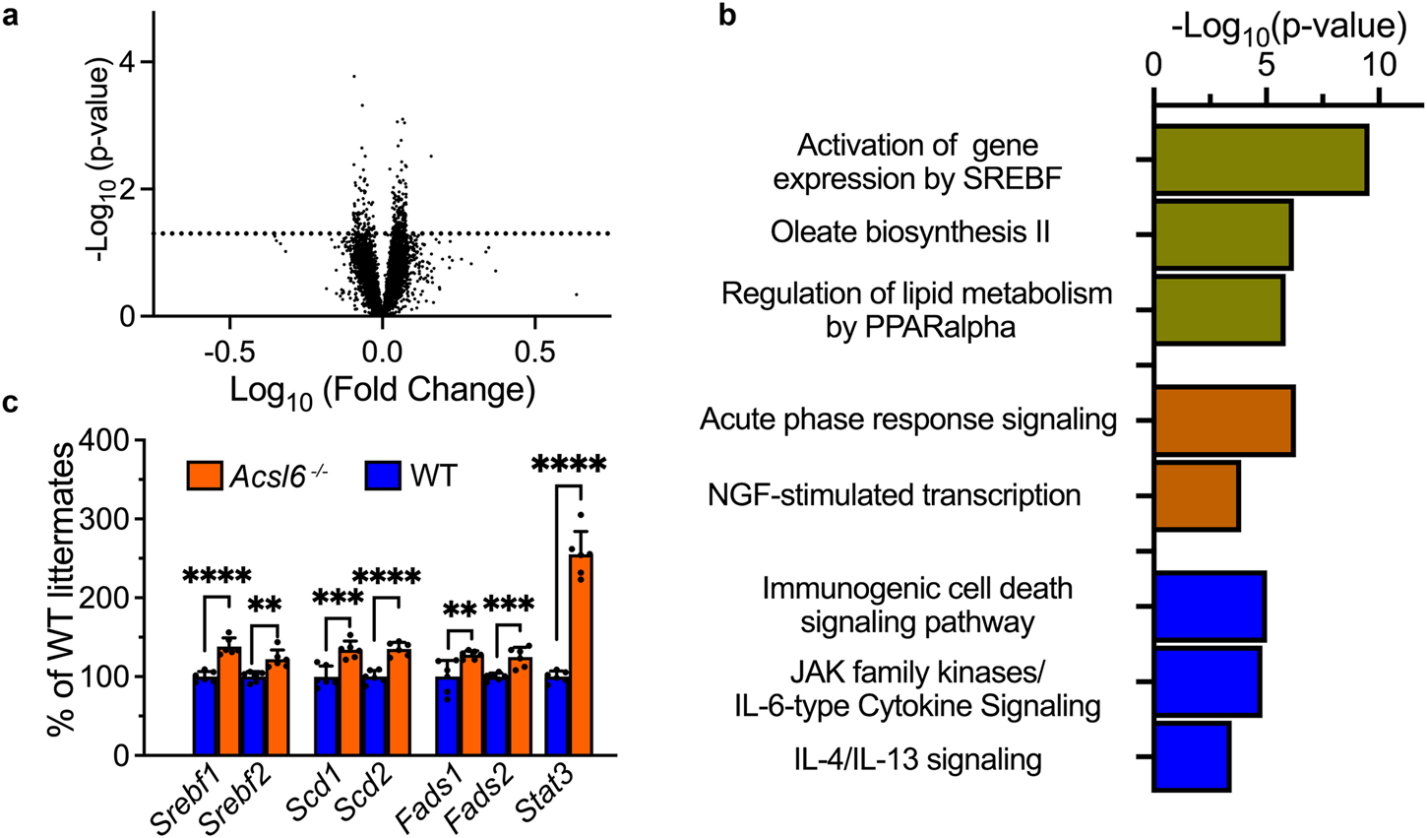
Transcriptional changes in the retinas of *Acsl6*^*−/−*^ mice. **a** Volcano plots showing differentially expressed genes in the retinas of *Acsl6*^*−/−*^ mice in comparison to WT littermate mice as detected with bulk RNAseq. **b** Top canonical pathways affected in *Acsl6*^*−/−*^ retinas were identified with the QIAGEN Ingenuity Pathway Analysis software. See also Table 1 and Supplementary Data 1. **c** Transcription analysis of selected genes in the retinas of 8-week-old *Acsl6*^*−/−*^ mice was performed using RT‒qPCR. Number of animals analyzed: *Acsl6*^*−/−*^ — 5, WT— 5. Data are shown as a percentage of average values for WT littermates and expressed as mean ± SD. *Srebf1/2* Sterol regulatory element binding transcription factor1/2,* Scd1/2* Stearoyl-CoA desaturase enzyme 1/2, *Fads1/2* Fatty acid desaturase 1/2, *Stat3* Signal transducer and activator of transcription 3.

The loss of DHA-containing phospholipids in the *Acsl6*^*−/−*^ retina was accompanied by compensatory changes which included elevated levels of phospholipids containing monosaturated and arachidonic fatty acids. These rearrangements could be explained by changes in the DHA transport to the retina but also alterations in the local synthesis of fatty acids behind BRB. Therefore, in the next set of experiments, we used comparative RNA-Seq analysis of whole retinas to explore the possibility of adaptational transcriptional changes accompanying phospholipid remodeling in *Acsl6*^*−/−*^ mice. After mapping RNAseq fastq-files to the mouse genome (Fig. 6a), we identified 184 differentially expressed genes in the retinas of *Acsl6*^*−/−*^ mice that were changed by at least 20%. The enrichment pathway analysis (Table 1 and Supplementary Data 1) showed elevated activity in “Activation of gene expression by SREBF”, “Oleate Biosynthesis II”, “Regulation of lipid metabolism by PPARα” as top pathways affected in the retinas of *Acsl6*^*−/−*^ mice (Fig. 6b, Table 1). Among genes assigned to these pathways were stearoyl-CoA desaturases 1 and 2 (*Scd1* and *Scd2*), fatty acid desaturases 1 and 2 (*Fads1* and *Fads2*), and sterol regulatory element-binding transcriptional factors 1/2 (*Srebf1/Srebf2)*^61–63^. An elevated expression of these genes in whole retinas was confirmed with RT-qPCR measurements (Fig. 6c). Other changes involved the *Stat3*-mediated stress signaling and inflammatory pathways commonly observed in many forms of retinal degeneration^64–66^. Stearoyl-CoA desaturases 1/2 act on saturated fatty acids to produce monounsaturated fatty acids^67,68^. Fatty acid desaturases 1/2 are indispensable for the biosynthesis of arachidonic fatty acid from simpler omega-6 fatty acid precursors^68,69^. Expression levels of these desaturases are controlled by protein products of *Srebf1/Srebf2* transcriptional factors^32,61,62,70^. Intriguingly, this transcriptional response is strikingly similar to the one previously observed in DHA-deficient *Mfsd2a*^*−/−*^ retinas, which suggests a possibility that this is a signatory transcriptional response to the DHA depletion caused by impaired DHA delivery to the retina/incorporation into the retinal membranes^9,10^. Such a response may explain, at least in part, the compensatory changes in the retinal lipid composition observed in the present study.

**Table 1:** Top canonical pathways affected the retinas of *Acsl6*^*−/−*^ mice

Biological processes	Ingenuity canonical pathways	-log_10_ (*p*-value)	Molecules
Lipid metabolism	Activation of gene expression by SREBF	9.6	*Cyp51a1, Dhcr7, Hmgcs1, Sc5d, Scd1, Sqle, Srebf1, Srebf2*
	Oleate Biosynthesis II	6.23	*Fads1, Fads2, Scd1, Scd2*
	Regulation of lipid metabolism by PPAR α	5.88	*Apoa1, Apoa2, Fabp1, Fads1, Hmgcs1, Med30, Srebf1, Srebf2*
Stress response	Acute Phase Response Signaling	6.33	*Apoa1, Apoa2, C1qa, C1qb, C1qc, Fos, Rbp3, Serpina1, Serpina3, Stat3*
	NGF-stimulated transcription	3.89	*Egr1, Fos, Junb, Trib1*
Inflammatory response	IL-4 / IL-13 signaling	5.05	*Anxa1, Bcl6, Cebpd, Fos, Hspa8, Junb, Stat3*
	JAK family kinases in IL-6-type Signaling	4.84	*Cebpd, Fos, Junb, Serpina1, Serpina3, Stat3*
	Immunogenic Cell Death Signaling Pathway	3.47	*Anxa1, Hspa1a/Hspa1b, Hspa1b, Hspa5, Hspa8*

Network analysis was performed on the list of the differentially expressed genes (Supplementary Data 1, tab “DE”) using QIAGEN Ingenuity Pathway Analysis Software with default parameters. See Supplementary Data 1 (tab “IPA”) for the full analysis results.

**Fig. 7:**
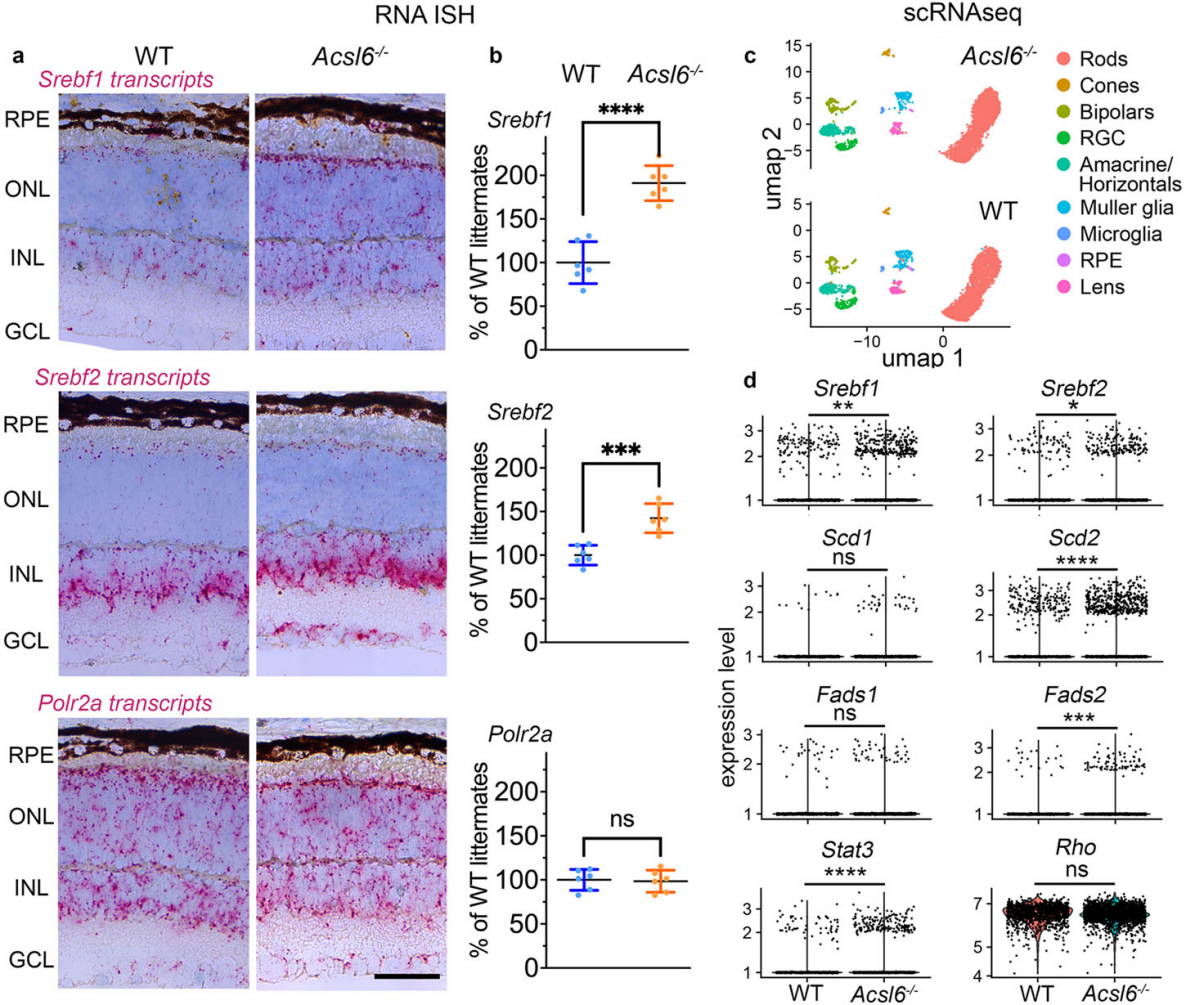
Elevated levels of *Srebf1/2* transcripts in *Acsl6*^*−/−*^ rods. **a** *Srebf1* and *Srebf2* transcripts in retinal cross-sections of indicated mouse genotypes were detected via RNA in situ hybridization (ISH). A probe for *Polr2a* (DNA-directed RNA polymerase II subunit RPB1) was used as a positive control to assess tissue quality. **b** Quantification of chromogenic signals in RNA ISH-stained sections in ONL areas of *Acsl6*^*−/−*^ (*n* = 6) and WT (*n* = 6) littermate mice. The data are presented as mean ± SD. ONL outer nuclear layer, INL inner nuclear layer, GCL ganglion cell layer, RPE retinal pigment epithelium. Scale bar, 50 μm. See also Supplementary Fig. 11 for examples of sections across an entire retina. **c** Uniform manifold approximation and projection (UMAP) plots of cells prepared from the retinas of 2-month-old *Acsl6*^*−/−*^ and WT mice. **d** Expression levels of selected genes in rod photoreceptor fractions of indicated mice (see also Supplementary Table 1). *Scd1/2* Stearoyl-CoA desaturase enzyme 1/2, *Fads1/2* Fatty acid desaturase 1/2, *Srebf1/2* Sterol regulatory element binding transcription factor1/2, Stat3 Signal transducer and activator of transcription 3. Rhodopsin (*Rho*) served as a control marker for rods.

In the next set of experiments, we sought to determine whether the *Srebf1/Srebf2* response is observed specifically in rods, the major DHA-containing cells in the retina. The RNA ISH analysis of wild-type retinas (Fig. 7a) showed different distributions of *Srebf1* and *Srebf2* transcripts. While *Srebf1* transcripts were found in all retinal layers, *Srebf2* transcripts were enriched in the inner retina. The levels of both *Srebf1* and *Srebf2* transcripts in the ONL, containing mostly rod photoreceptor nuclei, were low in comparison to those in other retinal layers. RNA ISH analysis of *Acsl6*^*−/−*^ retinas detected a pan-retinal increase in the staining of Srebf1/2 transcripts in all retinal layers, suggesting a coordinated response in all retinal cells, including rods (Fig. 7a, b, see also Supplementary Fig. 11). We next used single-cell RNA-seq (scRNA-seq) and a cell-selective strategy to validate *Srebf1/2* response specifically in *Acsl6*^*−/−*^ rods. The rod clusters were readily distinguishable in our uniform manifold approximation and projection (UMAP) plots of single-cell *Acsl6*^*−/−*^ and WT datasets (Fig. 7c). In agreement with the RNA ISH staining, we detected elevated transcripts for *Srebf1* and *Srebf2* levels, and their target desaturases in rod clusters. These changes were evident from both higher average values and an increased fraction of rods containing these transcripts (Fig. 7d, see also Supplementary Table 1). The limitations of scRNAseq sensitivity did not allow us to produce any additional interpretable results for rods or confidently assess changes in other retinal cells.

The modulation of *Srebf1/Srebf2* activity by PUFA has been documented in other experimental systems and is thought to restore membrane fluidity and structure upon global or specific lipid deprivation^32,71^. One may consider this transcriptional response (readily detected with either RT-qPCR or RNAseq) as an indicator of PUFA loss caused by impaired DHA delivery/retention in the retina.

## Discussion

In this study, we used a genetic approach to establish *Acsl6* as a major player in defining phospholipid composition and DHA retention in the retina. The most notable changes caused by *Acsl6* inactivation included: (1) a reduction in DHA-containing phospholipids; (2) a dramatic loss of VLC PUFA-containing phospholipids; and (3) a compensatory increase in MUFA- and AA-containing phospholipids. These changes differ from those observed in dietary studies in which DHA replacement with omega-6 docosapentaenoic acid (22:5 n-6) not naturally present in the retina and did not cause any major compensatory adjustments in phospholipids^24,25^. The analysis of *Acsl6*^*Retina KO*^ mice corroborated most of these findings and highlighted the role of the retinal *Acsl6* pool in the enrichment and retention of DHA behind the blood-retina barrier. Phospholipid changes in *Acsl6*^*−/−*^ mice resulted in pronounced defects in rod photoreceptor responses and progressive loss of these cells with age providing evidence in support of the biological significance of the specific phospholipid composition in photoreceptor function and viability.

One intriguing observation of this study is the similarity between the retinal phenotypes of lysolipid transporter *Mfsd2a*^*−/−*^ and *Acsl6*^*−/−*^ mice: the phospholipid composition in both mice is nearly identical, both show increased *Srebf1/Srebf2* transcriptional activity, their retinas have normal structure in younger mice but show progressive photoreceptor loss with age^8–10^. In combination with previous studies, our findings indicate that *Acsl6* and *Mfsd2a* are two potent regulators controlling DHA levels in both the brain and the retina^8–10,39^. They likely play complementary roles in DHA metabolism in neuronal tissues: while the lysolipid transporter Mfsd2a is responsible for DHA uptake from the blood, Acsl6 ensures efficient DHA incorporation into neuronal membranes. It is worth pointing out that an attribution of the retinal phenotype of *Mfsd2a*^*−/−*^ mice entirely to DHA loss was complicated by reports proposing that *Mfsda2* also functions as a negative regulator of transcytosis, a form of vesicular transport in brain and retina vasculature^72–74^. These studies correlated the developmental maturation of BRB and BBB with increased *Mfsd2a* expression and observed increased transcytosis in the neuronal vasculature of mice and zebrafish lacking *Mfsd2a*^72–74^. However, changes in BRB permeability were not observed by others in an alternative strain of *Mfsd2a*^*−/−*^ mice^9^. Other studies connected disruption of the BRB integrity with reduced expression of *Mfsd2a* in retinal vasculature under pathological conditions^75,76^. The mechanistic link connecting two functions of *Mfsd2a* as lysolipid lipid transporter and regulator of transcytosis in the neural vasculature remains under active investigation^77^. Future studies should establish the relative contributions of Acsl6 and Mfsd2a to defining phospholipid composition in neurons of the retina and the brain. *Acsl6* knockout mice have normal DHA levels in the liver^39,40^. Similar changes of phospholipid profiles in *Acsl6*^*Retina KO*^ and *Acsl6*^*−/−*^ mice corroborate the specific role of retinal *Acsl6* in DHA enrichment behind the blood-retina barrier in the absence of systemic changes. Finally, the interaction of *Acsl6* with DHA, its high affinity to DHA, and the cellular function of *Acsl6* are well-documented and understood. Therefore, *Acsl6*^*−/−*^ mice would serve as a potentially productive model to continue elucidating the characteristics of rod photoreceptor functioning and pathology caused by impaired DHA retention in the retina.

It has been postulated that high content of PUFAs in the retina is essential for supporting the formation of outer segment disc membranes, a process essential for maintaining photoreceptor functionality and health^1,3,44^. In contrast, our ultrastructural analysis shows that *Acsl6*^*−/−*^ rods have normal morphology despite a substantial loss of PUFAs, mirroring our previous observations in *Mfsd2a*^*−/−*^ mice^10^. Therefore, it is unlikely that rod photoreceptor loss in *Acsl6*^*−/−*^ mice is driven primarily by defects in the outer segment formation. Another hallmark of *Acsl6*^*−/−*^ mice is the accumulation of microglia/macrophages in the subretinal space before photoreceptor loss begins. The same phenomenon was observed in many other models of photoreceptor degeneration^41–43^. The presence of these cells was proposed to be beneficial under some pathological conditions, allowing the removal of dying cells, or detrimental in others^41,43^. Indeed, the presence of these mononuclear phagocytes could disrupt the microenvironment in subretinal space, impeding photoreceptor interaction with the RPE and driving pathological collapse. Interestingly, the anti-inflammatory impact of omega-3 PUFAs on microglia and macrophages is well documented in multiple in vitro and in vivo studies^78^. Therefore, it is reasonable to hypothesize that DHA depletion might switch microglia/macrophages to an inflammatory state, particularly in the subretinal space, since RPE and photoreceptors extensively exchange fatty acids. It will be interesting to investigate whether pharmacological or genetic approaches to hinder microglial migration could improve photoreceptor survival in *Acsl6*^*−/−*^ mice and to compare the transcriptional characteristics of immune cells found in the subretinal space of *Acsl6*^*−/−*^ mice and other models of photoreceptor degeneration^43,79^.

It has been further proposed that changes in the outer segment fatty acid composition may affect the biophysical properties of membranes and modify the efficiency of the phototransduction cascade functioning^1–4,54–59,80^. Mouse models with genetically disrupted DHA transport or retention in the retina provide a unique opportunity to address this long-standing question. The analysis of pharmacologically isolated rod light responses in retinas of young *Acsl6*^*−/−*^ mice demonstrated a major (~40%) functional deficit despite the lack of notable photoreceptor loss or overt morphological defects. In the context of retinal disease, these findings forecast compromised retinal function in various forms of retinal degeneration accompanied by DHA depletion or disturbances in DHA retention and supply in the retina. It is tempting to speculate that this phenotype directly reflects the change in phospholipid composition. Somewhat surprisingly, this effect was not phenocopied with single-cell recordings in DHA-deficient *Mfsd2a*^*−/−*^ mice^10^, which may suggest some of the consequences of *Acsl6* but not *Mfsd2a* loss are specific to photoreceptors. This will be addressed in future studies with rod-specific and double *Acsl6*/*Mfsd2a* knockout mice.

The DHA depletion in the retinas of both *Acsl6*^*−/−*^ and *Mfsd2a*^*−/−*^ mice was accompanied by an increase in the levels of *Srebf1/Srebf2* transcripts and target fatty acid desaturases genes controlled by *Srebf1/Srebf2* protein products. Although this transcriptional response was small (20–40%), it could have a physiological impact in vivo. The follow-up studies would investigate the functional role of these changes and whether they contribute to the observed compensatory remodeling of phospholipids and/or photoreceptor degeneration in DHA-deficient knockout retinas. The elevated levels of *Srebf1/2* transcripts and downstream components of the *Srebf1/2* pathway were also observed in the brains of DHA-deficient *Acsl6*^*−/−*^ and *Mfsd2a*^*−/−*^ mice^9–11,50^. The activation of the *Srebf1/2* pathway might be a universal neuronal stress response in both the retina and the brain counteracting DHA deficit. One may consider this response as a potential transcriptional indicator of changes in phospholipid composition in neuronal tissues caused by impairments in DHA transport and/or retention. Interestingly, SREBP1 induction was observed in the livers but not the brains of *Lpaat3*^*−/−*^ mice, which show DHA depletion in multiple tissues, including the liver, brain, and retina^31,32^. Mutation in the *Tmem135* gene also impacted the DHA levels in many tissues but triggered specific *Srebf1* upregulation in the eyecups^33,81^. Therefore, transcriptional responses in individual tissues caused by changes in DHA levels appear to depend on specific context of lipid metabolism disruption and could be a promising direction for future work.

While we were in the final stages of our manuscript preparation, a report describing another strain of whole-body *Acsl6* knockout mice was published^82^. Our findings align with this report, showing similar phospholipid changes, a lack of pronounced ultrastructural defects in photoreceptors, progressive photoreceptor loss, and reduced retinal function before the onset of measurable photoreceptor loss. Specific insights on the role of *Acsl6* in the retina identified in our study include the following. Our use of retina-specific *Acsl6* knockout mice enabled us to identify the role of the retinal pool of *Acsl6* in DHA retention behind the BRB in the absence of systemic changes in DHA synthesis, metabolism and transport. We used sensitive ex vivo ERG measurements to demonstrate that the functional defect in rods associated with changes in fatty acid composition of phospholipids precedes age-related retinal degeneration. We described and validated the lipogenic *Srebf1/2* transcriptional signature in the retina and rods and proposed its universal adaptational character as a response to DHA depletion. The similarity of the phospholipid profiles in *Acsl6*^*−/−*^ retinas to those found in DHA - deficient *Mfsd2a*^*−/−*^ mice and the lack of notable metabolic changes in *Acsl6*^*−/−*^ retinas strengthen our hypothesis that the observed phenotype in *Acsl6*^*−/−*^ mice could be attributed to the alterations in phospholipid composition and PUFAs depletion. Our study did not specifically investigate changes in cones.

Taken together, our studies have identified one of the critical players responsible for DHA retention in the retina and support the hypothesis that altered phospholipid composition of the retina could serve as the sole pathological driver or a contributing factor for rod photoreceptor degeneration and dysfunction. One puzzling observation is that photoreceptor loss in both *Acsl6*^*−/−*^ and *Mfsd2a*^*−/−*^ strains is slow, making it challenging to determine the precise mechanisms of photoreceptor death. One potential explanation is that, although reduced, the DHA levels in the retinas of these mice remain higher than those found in the blood (15–20% vs. 2–5%). Therefore, the retina likely possesses additional tissue-specific mechanisms for maintaining high DHA levels. Identifying these mechanisms might provide clues for the development of genetic mouse models with a deeper DHA deficit and more pronounced phenotypes, offering better tools to study the distinct roles of DHA in rod photoreceptor function and survival.

## Materials and methods

### Animals and animal procedures

Mice with floxed *Acsl6* gene, whole-body *Acsl6* and *Mfsd2a* knockout mice and *Chx10-Cre* transgenic mice were previously described^10,39,50,51^. Breeding schemes for all mouse lines and littermates used in experiments are shown in Supplementary Table 2. Animals were reared under a normal day/night cycle and handled according to protocols approved by the Institutional Animal Care and Use Committee of the University of Pittsburgh (#24014366). Mice were maintained on a chow diet with soy oil as a lipid source (Teklad Global 18% Protein Rodent Diet, 2918 Envigo). Mouse genotypes were determined using real-time PCR with specific probes designed for each gene (Transnetyx, Memphis, TN, USA). All experiments were performed using littermates as controls. Noninvasive experiments with animals (fundus and OCT) were performed as previously described^83,84^. Ex vivo ERG and isolation of photoreceptor responses were conducted as previously described^19,60^.

### Lipidomics and metabolomics

Analysis of phospholipids and total fatty acids extracted from the retina was performed on flash-frozen mouse retinas as described^15,33^. Metabolomics analysis was performed on flash-frozen retinas of mice fasted for 6 h as previously described^85,86^.

### Transcriptomics analysis and RNA ISH

RT‒qPCR was performed using primers listed in Supplementary Table 3 as previously described^83^. RNA ISH was performed on 5-μm-thick paraffin sections cut through the superior–inferior line prepared from formalin-fixed eyes using RNAscope probes (Advanced Cell Diagnostics, Hayward, CA, USA) listed in Supplementary Table 4, using an automated Leica Bond platform (Leica Microsystems GmbH, Wetzlar, Germany) following the manufacturer’s instructions. For quantification of *Srebf1/Srebf2* transcripts, the chromogenic dots on RNA ISH-stained sections were counted in 50 μm by 100 μm rectangles positioned around photoreceptor inner segments, the major area where an increase in straining was detected in *Acsl6*^*−/−*^ mice. *Polr2a* transcripts were quantified in the rectangles positioned in the middle of ONL, where individual chromogenic dots are easily visually separated. The measurements were performed in the superior part of the retina at 500 μm to 1000 μm distance from the optic nerve head. The results were expressed as % of the average measurements for the wild-type mice. The adjacent sections were stained for *Srebf1, Srebf2* and *Polr2a* transcripts for analysis.

Single-cell libraries for each genotype (*Acsl6*^*−/−*^ and WT) were prepared with pooled retinas from two-month-old male and female littermate mice using the 10x Chromium Platform following previously described protocols^10,87,88^. Libraries were prepared and data were analyzed together for both genotypes. Sequencing was performed at the UF Interdisciplinary Center for Biotechnology Research, and single-cell data analysis was performed as described in our previous studies^10,87^. Briefly, datasets were processed with the 10X Chromium Cell Ranger pipeline using default parameters, and further analyzed in RStudio with functions from Seurat and the functions from other packages specified below. Output files from Cell Ranger were transformed into Seurat objects. Cells containing more than 10% of mitochondrial genes, less than 200 and more than 20,000 counts per cell were considered damaged or clamped and were filtered out. Doublet cells were removed using scDblFinder function from the package of the same name. A correction for batch effects was done using Harmony function. After normalization, the individual groups of cells were identified using FindClusters function. The expression levels of Y-chromosome-specific (Eif2s3y, Uty, Ddx3y) and X-chromosome-specific genes (Xist, Tsix) were used to identify male and female cells. The following genes were used to identify cell clusters: ganglion cells (*Pou4f1*, *Pou4f2*, *Rbpms*), amacrine and horizontal cell cells (*Tfap2a*, *Pax6*, *Gad1*, *Onecut2*, *Calb1/2*), bipolar cells (*Vsx2*, *Nyx*, *Prkca*, *Grm6*, *Sebox*), rod (*Nrl*, *Nr2e3*) and cone (*Opn1mw*, *Arr3*) photoreceptors, Muller glia (*Aqp4*, *Glul*, *Pax2*, *S100b*, *Rlbp1*), RPE (*RPE65*, *Lrat*), inflammatory cells (*Aif1*, *Tmem119*, *Tlr4*), lens/fibroblast contamination (*Grifin*, *Mip*, *Cryba1*). Differential expression analysis was performed using FindMarkers function on female rod clusters, which were selected with CellSelector function.

For whole-retina sequencing, total RNA was prepared from the retinas of six *Acsl6*^*−/−*^ and four WT (*Acsl6*^*+/+*^) 8-week-old female mice using the RNeasy Mini Kit (Qiagen) with DNase digestion (RNase-Free DNase Set, Qiagen). Retinas were collected between 1:00 and 2:00 P.M. from mice killed with isoflurane, carefully dissected under a microscope and snap-frozen in liquid nitrogen. The libraries were prepared and sequenced at GENEWIZ. FASTQ files were aligned to mouse genome GRCm39 using DNAStar software v15.3.0 and normalized by reads per kilobase (RPKM). Genes expressed at the level exceeding 10 RPKM of base average for all samples were filtered out in ArrayStar and further analyzed in Prism GraphPad software. Genes that changed for >20% with *p* < 0.05 (as determined Benjamini–Hochberg test with FDR 5% threshold) were considered differentially expressed. Fold-change in the expression of genes was calculated based on the mean values for *Acsl6*^*−/−*^ and WT littermates. A list of differentially expressed genes in *Acsl6*^*−/−*^ mice is presented in Supplementary Data 1 Excel file (sheet “DE”). To establish cellular pathways affected by *Acsl6* loss, differentially expressed genes were analyzed with the QIAGEN Ingenuity Pathway Analysis Software (v111725566) based on the fold-ratio using the default parameters. Enriched pathways with *p* < 0.05 are listed in the Excel file Supplementary Data 1 (sheet “IPA”). The RNA-seq datasets are deposited at NCBI (Bioproject PRJNA1013575).

**Histology and immunolocalization**. Morphometric analysis was performed on 0.5-μm-thick resin sections cut along the superior–inferior line of the eye containing the optic nerve. The sections were stained with toluidine blue and quantified as previously described^89^. The preparation of sections was performed as previously described^90,91^. Iba1-positive cells were detected in retinas fixed in a 4% PFA-PBS solution. Following 1 h of fixation at room temperature, the retinas were washed in PBS and incubated with a blocking solution (PBS, 5% donkey serum, 0.3% Triton X) for 1 h at room temperature. Next, the retinas were incubated overnight in a cold room with rabbit anti-Iba1 antibody (019-19741, Wako Chemicals, Richmond, VA, USA) diluted 1:500 in the blocking solution. After extensive washing with PBS supplemented with 0.3% Triton X, the retinas were incubated with Alexa Fluor™ 555 goat anti-rabbit antibody (A21429, Invitrogen, Waltham, MA, USA) at a 1:1000 dilution for 1 h at room temperature. The retinas were then washed, flat mounted, coverslipped with Fluoromount-G™ Mounting Medium (00-4958-02, Thermo Fisher, Waltham, MA, USA), and visualized using a Leica TCS SP8 confocal microscope.

### Western blotting

Western blotting was performed using previously described protocols^83,84^. Anti-ACSL6 (HPA040470, Sigma), anti-β-ACTIN (Invitrogen, BA3R, MA5-15739-D800), anti-CRE (Invitrogen, PA5-32244) and secondary 680 anti-rabbit antibodies (Jackson ImmunoResearch, 711-625-152) were used for detection. Protein bands were visualized with the Odyssey Infrared Imaging System (LI-COR Biosciences, Lincoln, NE, USA). Full blot images are shown in Supplementary Fig. 9.

### Statistics and reproducibility

Differences were considered statistically significant when *p* < 0.05, as determined by the two-tailed Student’s test using GraphPad Software with Benjamini–Hochberg multiple comparison correction if specified in figure legends or in supplementary materials and datasets. The *p* values in the figures are indicated as follows: *p  <  0.05, **p  <  0.01, ***p  <  0.001, ****p  <  0.0001, and ns, for *p*  >  0.05.

### Supplementary information

Supplementary Materials

Description of additional supplementary file

Supplementary Data 1.

Supplementary Data 2.

## Data Availability

All data needed to evaluate the conclusions of this study are presented in the paper, Supplementary Figs., Tables, and data files. Supplementary Data 1 file contains the results of gene expression analysis. Supplementary Data 2 file contains the source data displayed in the plots on the figures of main text. RNA-seq data have been deposited at NCBI (Bioprojects PRJNA1013575 and PRJNA948700). Detailed experimental protocols and data analysis workflows are available from the corresponding author upon reasonable request.
