# Supplementary material for: Acyl-CoA synthetase 6 controls rod photoreceptor function and survival by shaping the phospholipid composition of retinal membranes
Source: Commun Biol. 2024 Aug 21;7:1027. doi: 10.1038/s42003-024-06691-8 (PMC11339274; doi:10.1038/s42003-024-06691-8)
Supplement: Supplementary file 1 — Supplementary Materials [file 42003_2024_6691_MOESM1_ESM.pdf]

Supplementary Materials for

Acyl-CoA synthetase 6 controls rod photoreceptor function and survival by shaping the phospholipid composition of retinal membranes

Yixiao Wang[1#], Silke Becker[2#], Stella Finkelstein[3], Frank M. Dyka[4], Haitao Liu[4], Mark Eminhizer[5], Ying Hao[3], Richard S Brush[6], William J. Spencer[7], Vadim Y. Arshavsky[3], John D. Ash[4], Jianhai Du[5], Martin-Paul Agbaga[6], Frans Vinberg[2], Jessica M. Ellis[8], Ekaterina S. Lobanova[4*]

[1]Department of Ophthalmology, University of Florida, Gainesville, FL, USA

[2]Department of Ophthalmology, University of Utah, Salt Lake City, UT, USA

[3]Department of Ophthalmology, Duke University, Durham, NC, USA

[4]Department of Ophthalmology, University of Pittsburgh, Pittsburgh, PA, USA

[5]Departments of Ophthalmology and Visual Sciences and Biochemistry and Molecular Medicine, West Virginia University, Morgantown, WV, USA

[6]Department of Ophthalmology, University of Oklahoma Health Sciences Center and Dean McGee Eye Institute, Oklahoma City, OK, USA

[7]Department of Ophthalmology and Visual Sciences, SUNY Upstate Medical University, Syracuse, NY, United States, USA

[8]East Carolina University, Greenville, NC, USA

*****Correspondence: lobanova@pitt.edu (ESL)

#These authors contributed equally

Ekaterina Lobanova, PhD
Associate Professor
Department of Ophthalmology
University of Pittsburgh
1622 Locust St
Rm 7.392
Pittsburgh PA
15219
Phone: 1(412) 648-3906

Supplementary Figures 1 to 11
Supplementary Tables 1 to 4
Legends for Supplementary Data

Other Supplementary Material:

Supplementary Data 1
Supplementary Data 2

Supplementary Fig. 1. *Acsl6* and *Polr2a* transcripts in the retinas of pigmented (C57BL/J) and albino (BALB/CJ) wild-type mice detected with RNA in situ hybridization (RNA ISH).

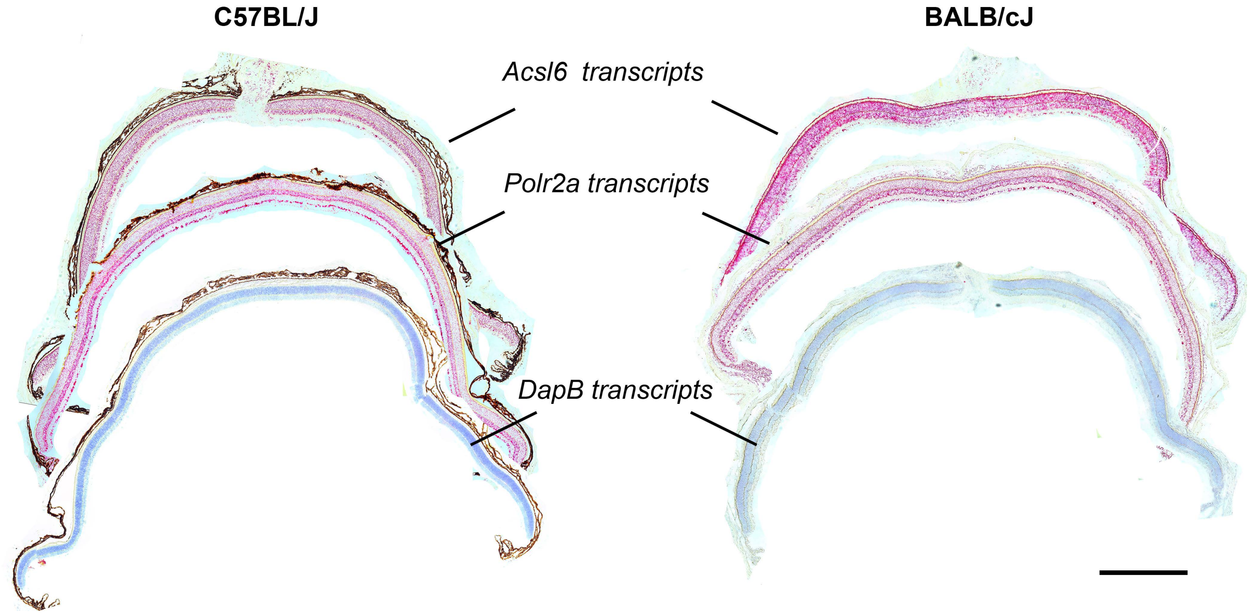

RNA ISH analysis of retinal sections cut along an entire eye (inferior – superior axis from left to right). The signals for *Acsl6* or *Polr2a* transcripts appear as red puncta. Samples were processed together using the same conditions. Representative regions of the cross-sections are shown in **Fig. 1a** in the main text. A *DapB* probe detected scarce signal and was used as negative control. All mice were one-month-old. The scale bar is 500 μm.

Supplementary Fig. 2. Characterization of rod photoreceptor loss in *Acsl6*$^{-/-}$ and *Acsl6*$^{Retina\ KO}$ mice.

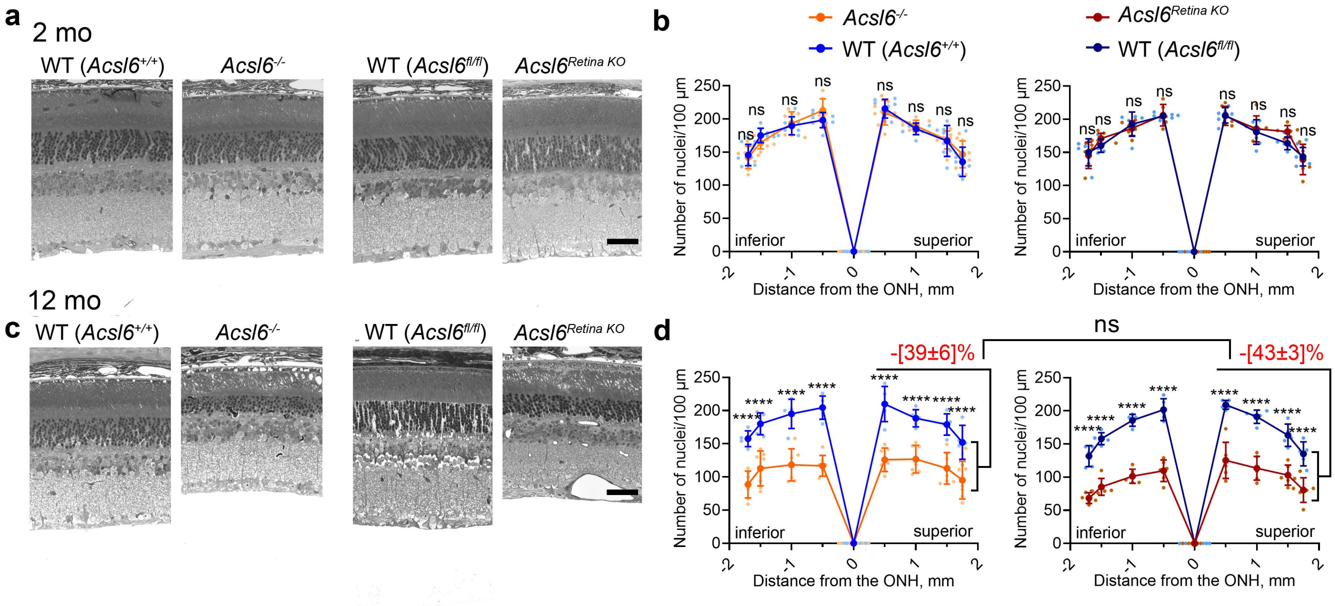

Morphometric analysis of retinas from 2-month-old **(a, b)** and 12-month-old **(c, d)** mice of the indicated genotypes. **(a, c)** Images of the representative regions of toluidine blue-stained sections from the superior parts of the retina are shown in grayscale. Scale bar, 50 µm. **(b, d)** Spider diagrams show the number of nuclei in 100-µm segments counted along the inferior – superior axis at different distances from ONH (Optic Nerve Head) in the mouse retinas in ONL (Outer Nuclei Layer). The cross sections cut through the entire retina are shown in **Supplementary Fig. 3**. The number of eyes analyzed was in 2 mo mice: *Acsl6*$^{-/-}$ — 8, WT (*Acsl6*$^{+/+}$) — 8, *Acsl6*$^{Retina\ KO}$ — 6, WT (*Acsl6*$^{fl/fl}$) — 6, in 12 mo mice: *Acsl6*$^{-/-}$ — 7 and WT (*Acsl6*$^{+/+}$) — 5, *Acsl6*$^{Retina\ KO}$— 6 and WT (*Acsl6*$^{fl/fl}$) — 5. The difference in the extent of nuclei loss in the outer retinas of 12-month-old *Acsl6*$^{-/-}$ and *Acsl6*$^{Retina\ KO}$ mice was not statistically significant. The data are presented as the mean ± SD. Quantification was performed by individuals not aware of the genotypes.

Supplementary Fig. 3. Representative retinal sections across an entire retina in *Acsl6*$^{-/-}$ and *Acsl6*$^{Retina\ KO}$ mice at different ages.

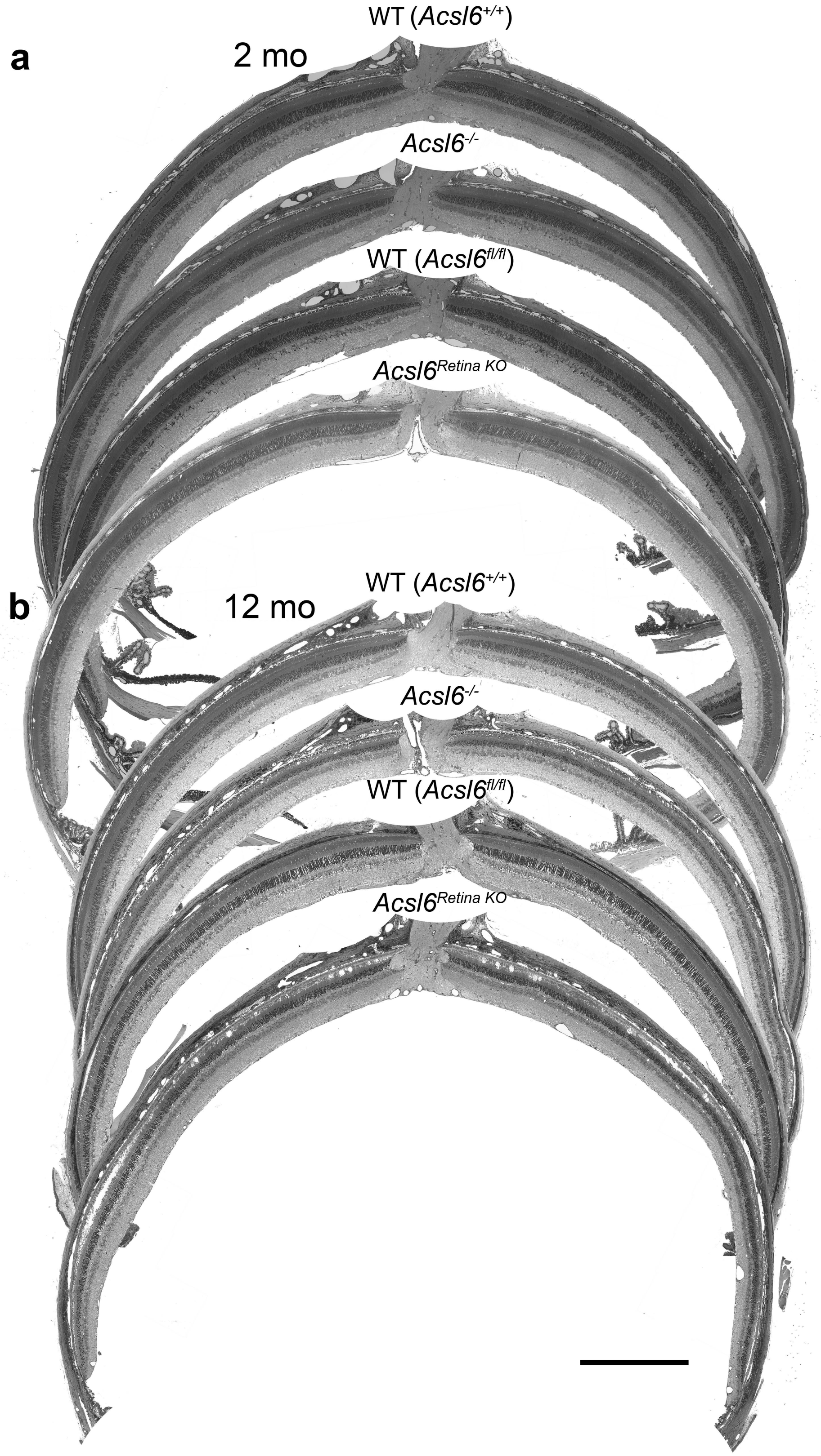

(a, b) Toluidine blue staining of 0.5-µm-thick resin sections cut along inferior – superior axis of the eye (left to right) from **(a)** 2-month-old and **(b)** 12-month-old *Acsl6*$^{-/-}$ and *Acsl6*$^{Retina\ KO}$ mice and their WT littermates at the indicated ages. Sections are shown in grayscale. Scale bar, 500 µm. Representative regions of the cross-sections from the panel are shown in **Supplementary Fig. 2ac**.

Supplementary Fig. 4. Accumulation of Iba1-positive cells in the outer segment area of *Acsl6*$^{-/-}$ mice.

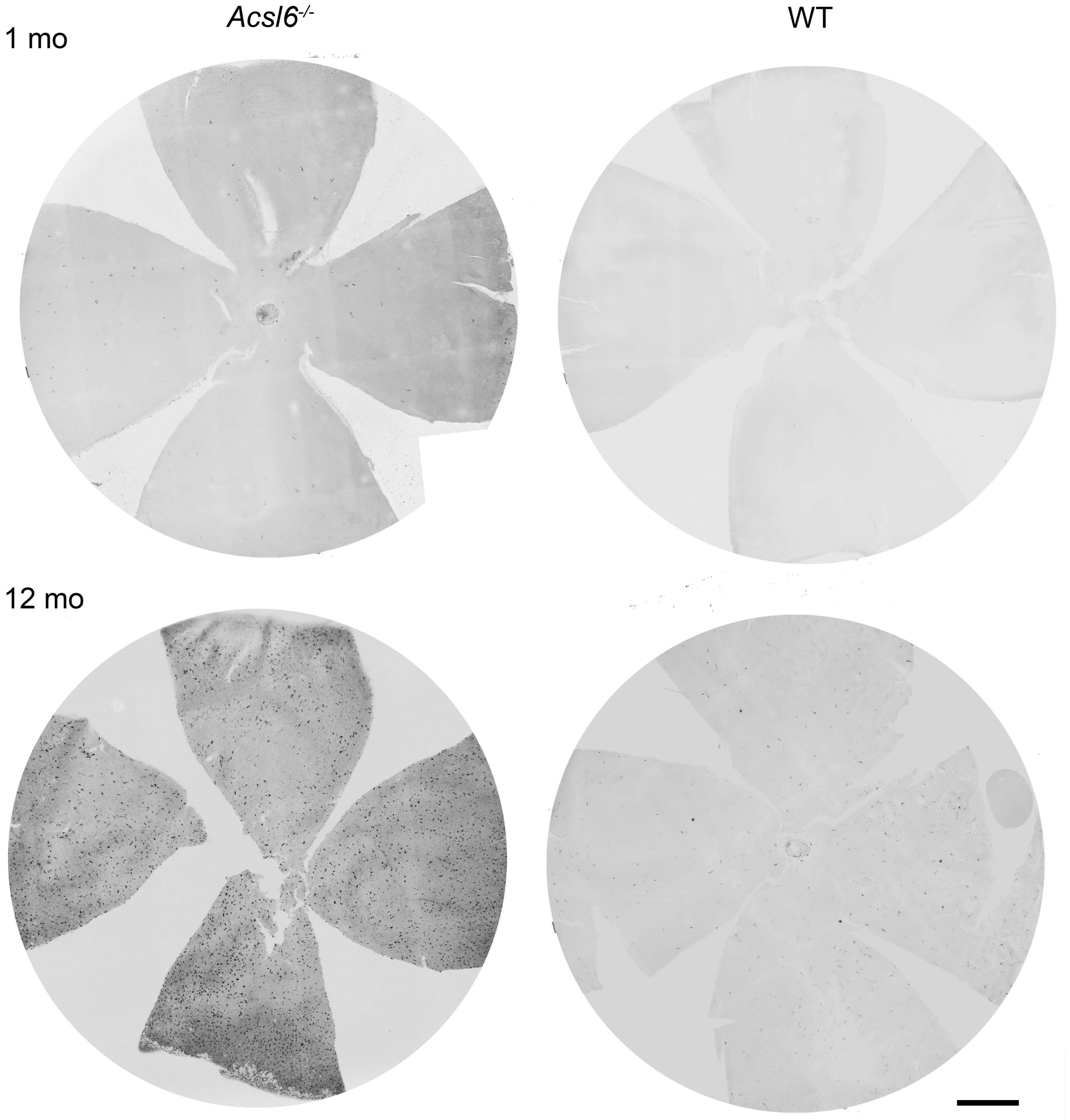

Staining for Iba1-positive cells in the subretinal space on retinal flat mounts from 1-month-old and 12-month-old *Acsl6*$^{-/-}$ and WT littermate mice. Z-stack images in the outer segment areas were collected using confocal microscopy and collapsed with a maximum projection function. An inverted grayscale image is shown. Scale bar: 500 µm.

Supplementary Fig. 5. Ultrastructural analysis of photoreceptor outer segments in *Acsl6*$^{-/-}$ mice.

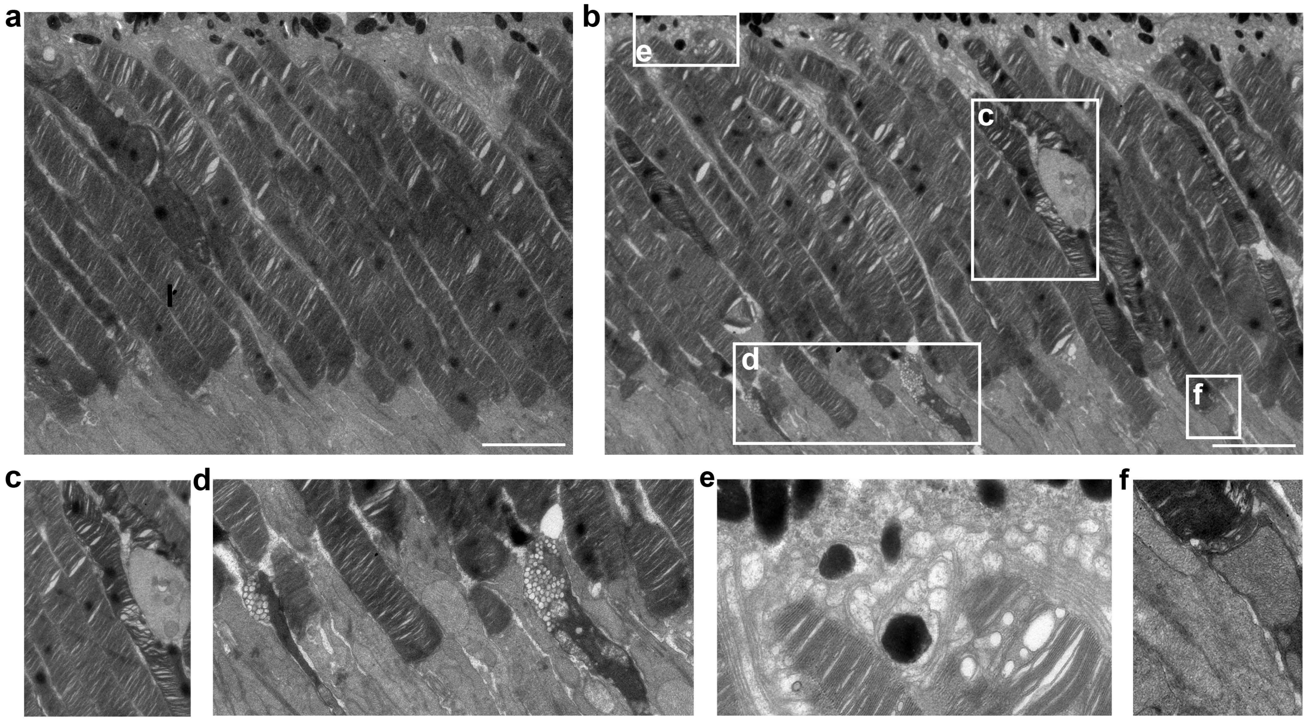

(a) Normal-appearing photoreceptor outer segments and **(b)** occasional structural abnormalities observed in the outer segment areas of 9-month *Acsl6*$^{-/-}$ mice. Scale bars are 10 µm. **(c-f)** Magnified insets from panel **(b)**: **(c)** Distorted and raptured outer segments next to an immune cell, **(d)** vesiculating inner segments of dying photoreceptors, **(e)** vesicular material between RPE and the tips of the outer segments, **(f)** swollen mitochondria in the rod photoreceptors.

Supplementary Fig. 6. Analysis of changes in the basal levels of representative metabolites in the retinas of *Acsl6*$^{-/-}$ mice in comparison to WT littermates.

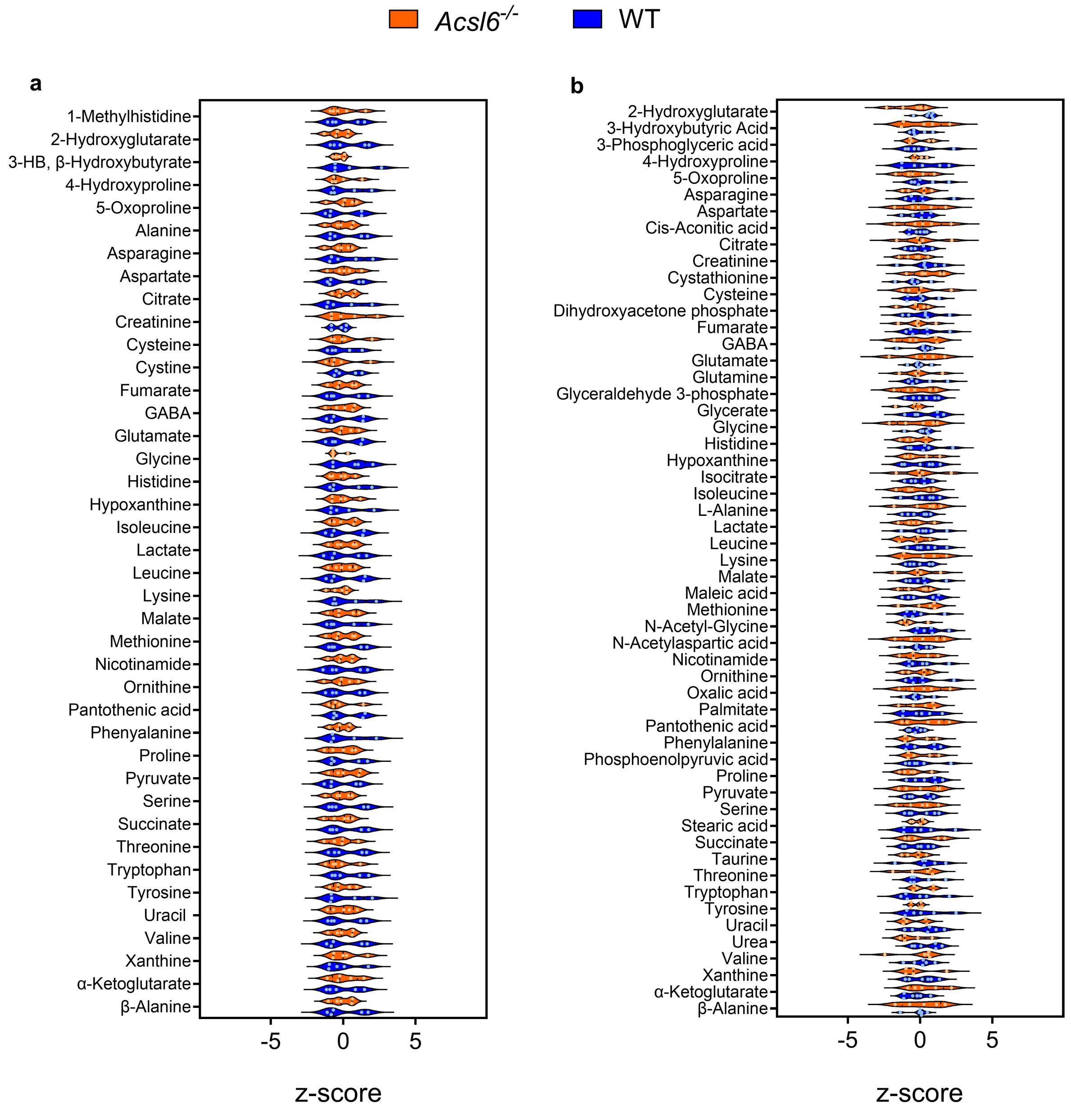

Violin plots for z-scores for the representative metabolites in the retinas of **(a)** 2-month-old (*Acsl6*$^{-/-}$ — 5, WT (Acsl6$^{+/+}$) — 5) and **(b)** 6-month-old (*Acsl6*$^{-/-}$ — 5 and WT(Acsl6$^{+/+}$) — 6) mice. None of the changes reached the level of statistical significance.

Supplementary Fig. 7. Comparison of age-related changes in retinal structure in *Acsl6*$^{-/-}$ and *Acsl6*$^{Retina\ KO}$ mouse lines as detected by *in vivo* imaging techniques.

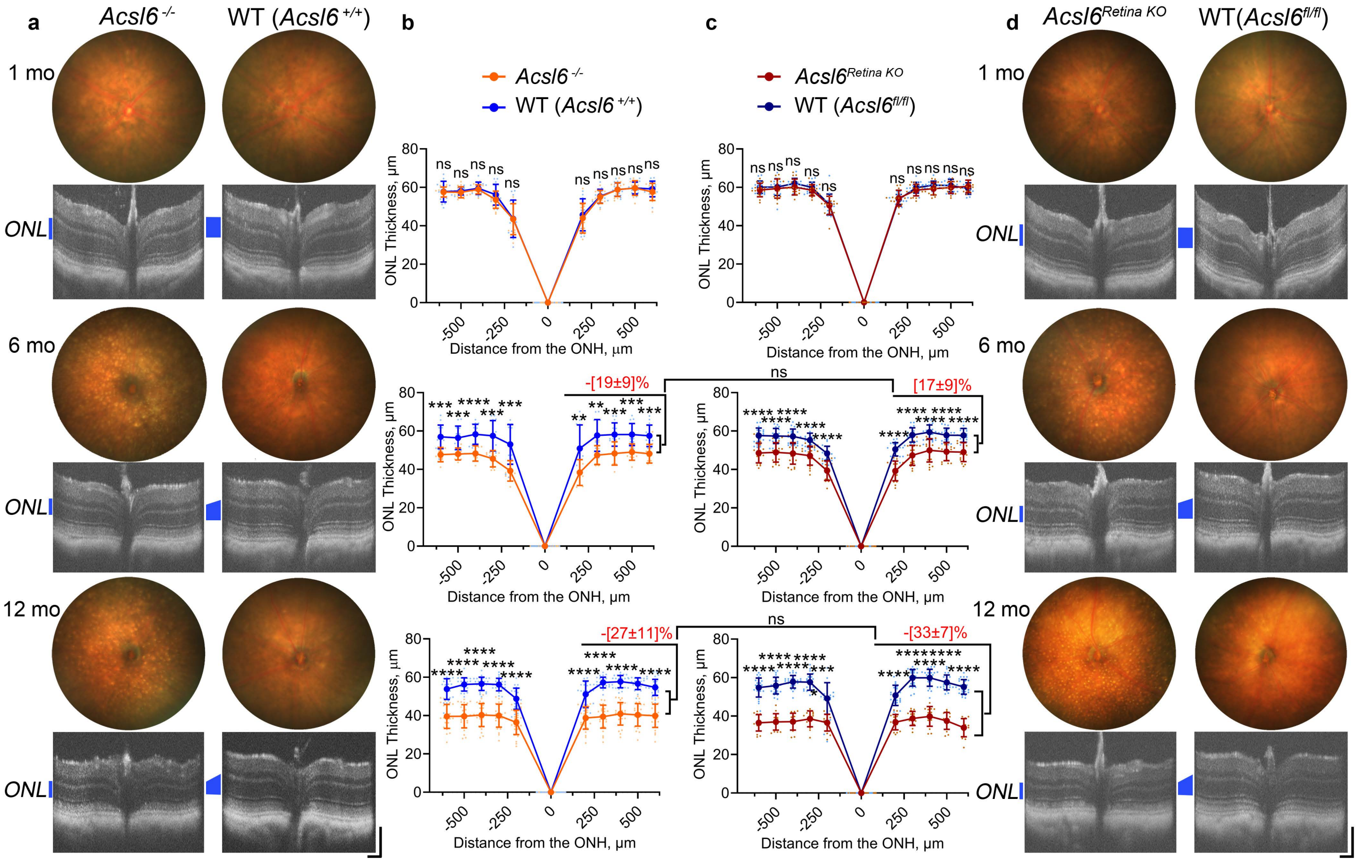

a, d Representative funduscopic images and horizontal SD-OCT scans of mice with the indicated genotypes at different ages (temporal-nasal from left to right). **b, c** Horizontal OCT spider diagrams of the outer nuclear layer (ONL) thickness for mice with the indicated genotypes at 1, 6 and 12 months of age. The ONL is marked with a blue vertical line on OCT images; scale bar, 100 µm. To estimate ONL thickness changes, measurements from OCT spider diagrams for each mouse were summed and expressed as a percentage of the average value calculated for WT littermates. The number of eyes analyzed was as follows: 1 mo, *Acsl6*$^{-/-}$ — 13 and WT — 13, *Acsl6*$^{Retina\ KO}$ — 14 and WT (*Acsl6*$^{fl/fl}$) — 15; 6 mo, *Acsl6*$^{-/-}$ — 10 and WT — 14, *Acsl6*$^{Retina\ KO}$—19 and WT (*Acsl6*$^{fl/fl}$) — 20, 12 mo, *Acsl6*$^{-/-}$ — 26 and WT — 24, *Acsl6*$^{Retina\ KO}$ — 15 and WT (*Acsl6*$^{fl/fl}$) — 24. The data are presented as mean ± SD. Quantification was performed by individuals not aware of specific genotypes. Panels **a** and **b** are also shown in **Fig. 2** in the main text. The differences in the extent of age-related ONL thinning between *Acsl6*$^{-/-}$ and *Acsl6*$^{Retina\ KO}$ mouse were not statistically significant.

Supplementary Fig. 8. Comparison of phospholipid changes in retinas of *Acsl6*$^{-/-}$ and *Acsl6*$^{Retina\ KO}$ mice.

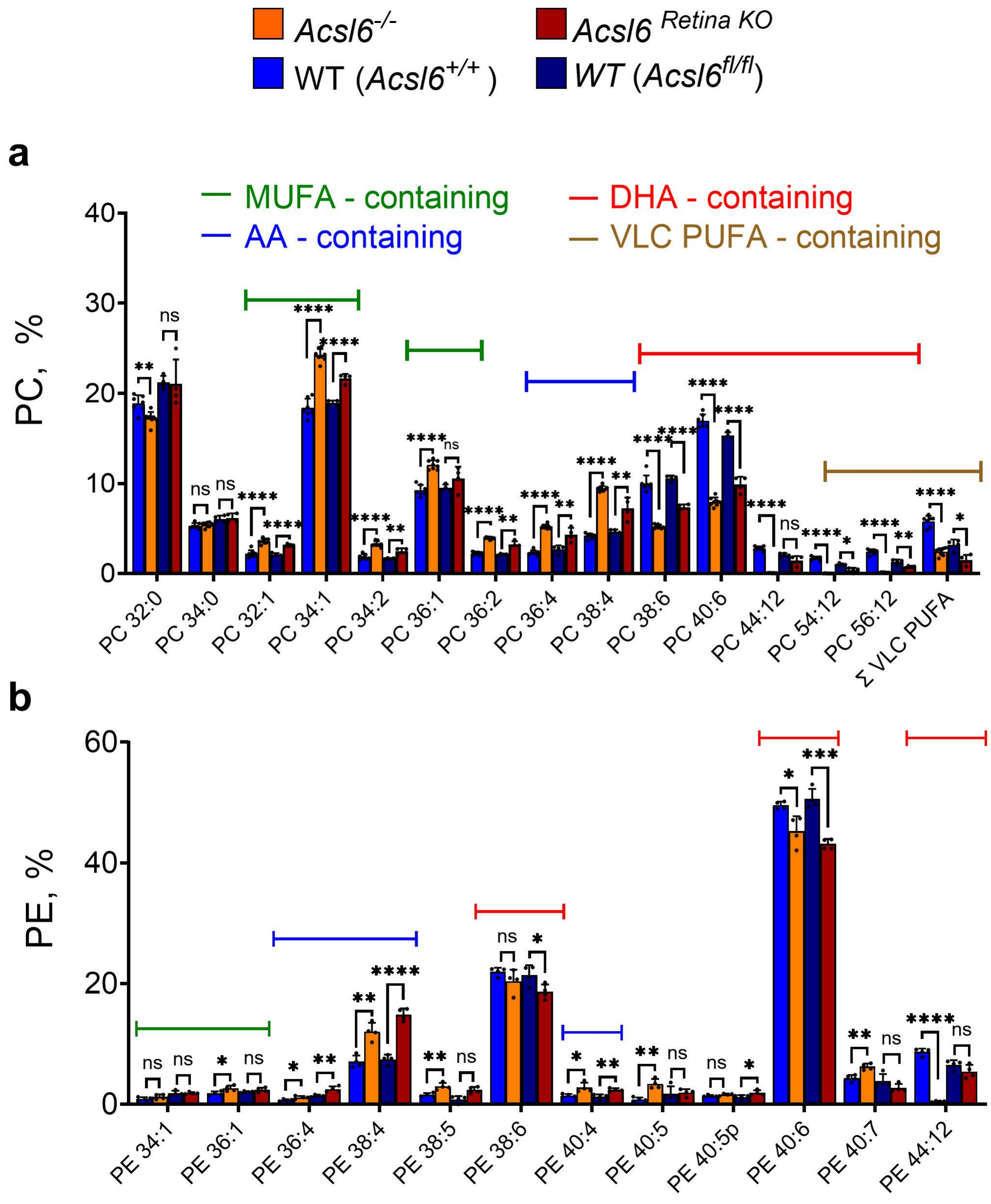

Profiles for (**a**) phosphatidylcholine and (**b**) phosphatidylethanolamine phospholipids are shown as percentages within the corresponding class. The number of samples used for phospholipid analysis was as follows: *Acsl6*$^{-/-}$— 4 and WT (*Acsl6*$^{+/+}$) — 4; *Acsl6*$^{Retina\ KO}$ — 4 and WT (*Acsl6*$^{fl/fl}$) — 4. MUFA, monounsaturated fatty acid; AA, arachidonic fatty acid; DHA, docosahexaenoic fatty acid; VLC PUFA, very-long-chain polyunsaturated fatty acid. The analyzed mice were two-month-old. The data are presented as mean ± SD.

Supplementary Fig. 9. Comparison of the ACSL6 protein loss in the retinas of *Acsl6*$^{-/-}$ and *Acsl6*$^{Retina\ KO}$ mouse lines.

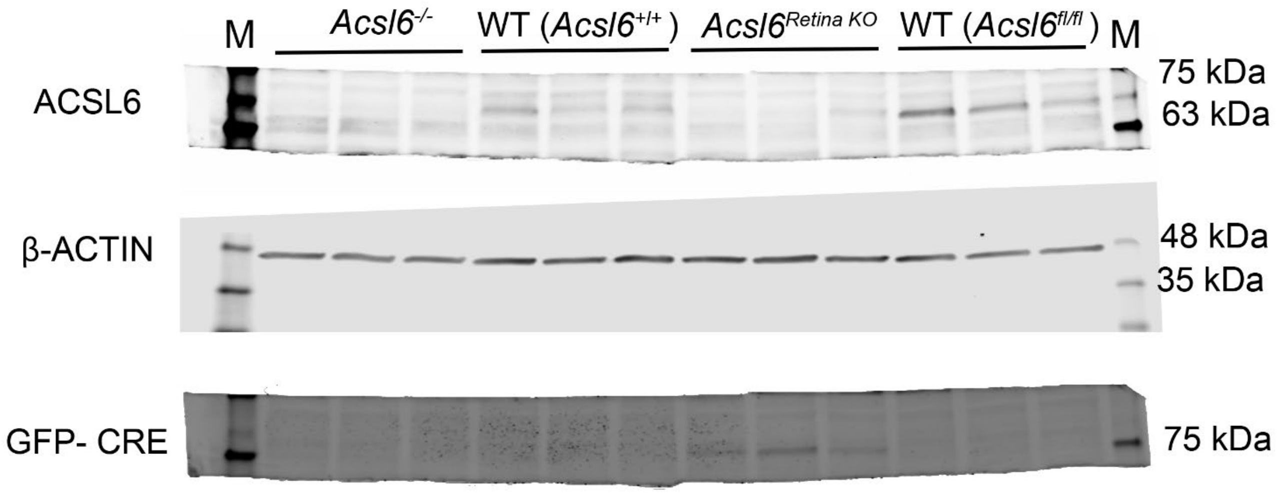

Western blot for ACSL6 protein in the retinas of 1-month-old mice, as indicated. β-ACTIN was used as loading control. GFP-CRE fusion protein served as a marker to identify *Chx10-Cre* transgenic mice. The left portions of the western blot panels are also shown in **Fig. 1c** of the main text.

Supplementary Fig. 10. *Ex vivo* ERG rod responses continue to decline with age in *Acsl6*$^{-/-}$ knockout mice.

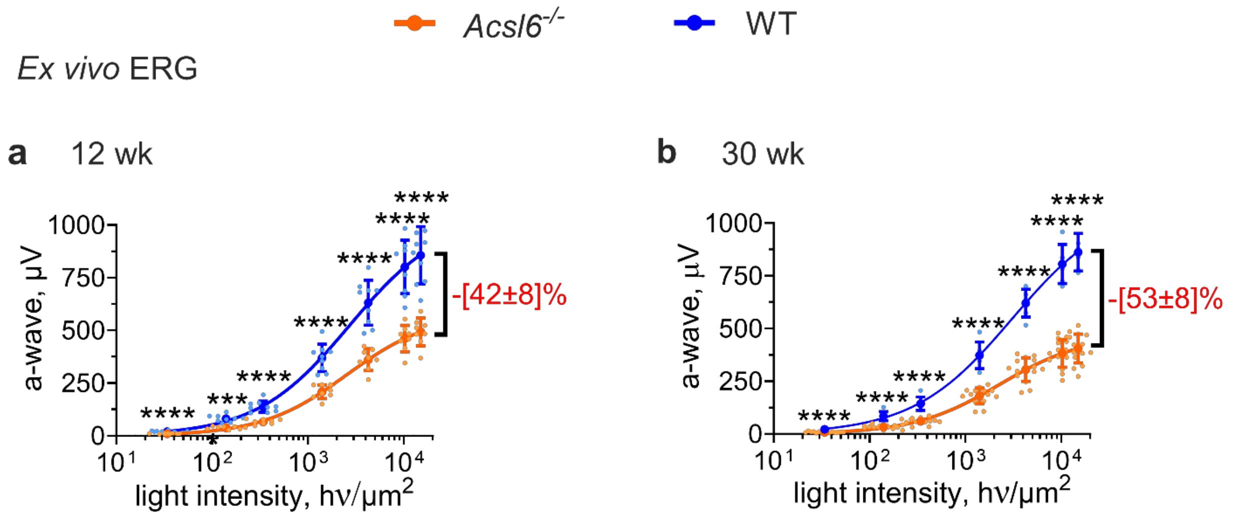

Comparison of pharmacologically isolated light responses from rod photoreceptors measured with *ex vivo* ERG in the retinas of (**a**) 12-week-old mice and (**b**) 30-week-old mice. The number of retinas: 12 wk (*Acsl6*$^{-/-}$ — 8, WT— 9) and 30 wk (*Acsl6*$^{-/-}$ — 15, WT— 6). Reduction in responses at maximum light intensity was computed as percentage to the average values for wild type littermate mice. The data are presented as mean ± SD. Panel **a** is also shown in **Fig. 5** of the main text.

Supplementary Fig. 11. *Srebf1*, *Srebf2* and *Polr2a* transcripts in the retinas of *Acsl6*$^{-/-}$ and wild-type mice as detected with RNA in situ hybridization (RNA ISH).

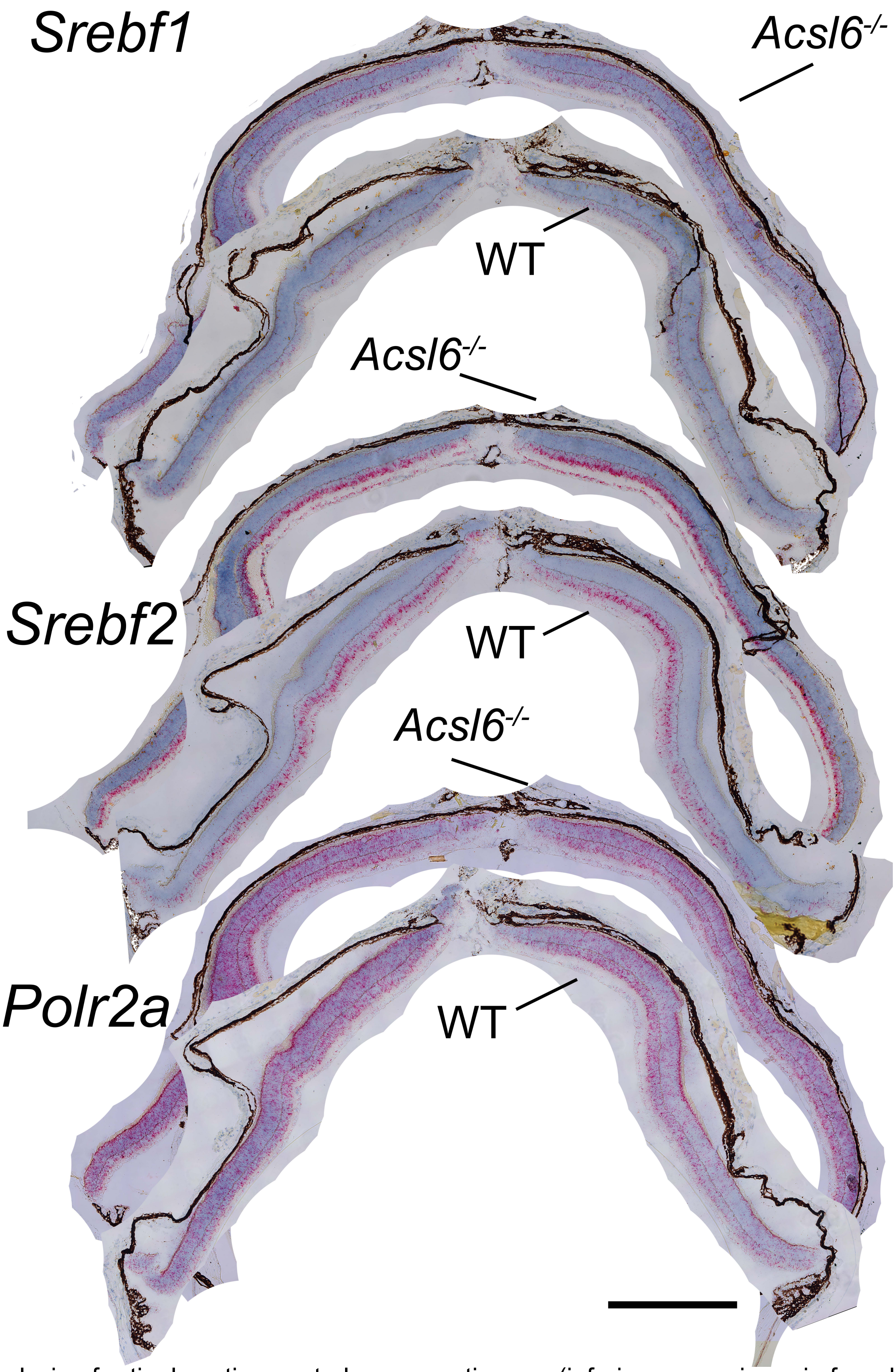

RNA ISH analysis of retinal sections cut along an entire eye (inferior – superior axis from left to right). Transcript signals appear as red puncta. Samples were processed together using the same conditions. Representative regions of the cross-sections from superior part are shown in **Fig. 7a**. The scale bar is 500 μm.

Supplementary Table 1. Changes in the levels of representative transcripts in *Acsl6$^{-/-}$* *and* WT rod clusters of scRNAseq dataset. Fractions of cells expressing the gene of interest, fold-changes in the average expression levels of genes of interest, and P-values were calculated using FindMarkers function.

	Fraction of wild type rods expressing indicated gene	Fraction of *Acsl6$^{-/-}$* rods expressing indicated gene	Fold change in the fraction of rods expressing indicated gene (*Acsl6$^{-/-}$* vs WT rods)	Fold change in the average expression level of indicated gene (*Acsl6$^{-/-}$* vs WT rods)	P-value
Srebf1	0.117	0.159	1.36	1.32	0.00224
Srebf2	0.075	0.099	1.32	1.27	0.02885
Scd1	0.010	0.020	2	1.63	0.07244
Scd2	0.179	0.244	1.36	1.36	4.31E-05
Fads1	0.025	0.025	1	1.09	0.75883
Fads2	0.020	0.046	2.3	1.82	0.00096
Rho	1	1	1	0.99	0.91962
Stat3	0.052	0.099	1.9	1.97	9.54E-07

Supplementary Table 2. Breeding strategies and littermates used in experiments. NA: not applicable

Line abbreviation	Breeding strategy to establish maintain the line or lines used	Littermates used in the experiments
WT (C57BL/6J)	Jackson Lab (Stock# 000664)	NA
WT (BALB/cJ)	Jackson Lab (Stock# 000651)	NA
Acsl6$^{-/-}$	*Acsl6*$^{+/-}$ × *Acsl6*$^{+/-}$	*Acsl6*$^{-/-}$ vs. WT
Acsl6$^{Retina\ KO}$	*Acsl6*$^{fl/fl}$*/Chx10-Cre(+)* × *Acsl6*$^{fl/fl}$	*Acsl6*$^{fl/fl}$ */Chx10-Cre(+)* vs. *Acsl6*$^{fl/fl}$
Mfsd2a$^{-/-}$	*Mfsd2a*$^{+/-}$ × *Mfsd2a*$^{+/-}$	*Mfsd2a*$^{-/-}$ vs. WT

Supplementary Table 3. RT–qPCR primer sets.

Gene	Forward Primer	Reverse Primer
Actin	*cgagcacagcttctttgcag*	*ttcccaccatcacaccctgg*
Fads1	*tgcaacgttcaccaatcagc*	*gggattgtactaggggtgcc*
Fads2	*acacagatgaaccaccttgtca*	*tctggaaattgaggtgcccg*
Scd1	*tgacctgaaagccgagaagc*	*aggtgctaacgaacaggctg*
Scd2	*tgtctgacctgaaagccgag*	*tgtttacaaaagtctcgcccc*
Srebf1	*caggttccagtggcaaagga*	*ccaagcttctctacggtgcg*
Srebf2	*cagccctacccgtacacac*	*ggaagacaggaaagagagggg*
Stat3	*accaacgacctgcagcaata*	*acactccgaggtcagatcca*

Supplementary Table 4. RNA in situ hybridization (RNA ISH) probes were used to visualize RNA transcripts and as controls on paraffin-embedded retinal sections.

Gene	Reference number	Lot number	Manufacturer
Mm-Acsl6-O1-C1	1205338-C1	22237B	Advanced Cell Diagnostics, Inc.
Mm-Srebf1	562268	23060A	
Mm-Srebf2	416018	23060A	
Mm-Polr2a (positive control)	312478	22237B	
DapB (negative control)	312038	22236A	

Supplementary Data 1. (Excel Microsoft file

Tab "DE"

Differentially expressed genes in the retinas of *Acsl6*$^{-/-}$ mice in comparison to WT littermates were identified via bulk RNAseq. P-values represent a false discovery rate. Genes with p-values <0.05 and changed by at least 20% were considered differentially expressed. Log10 [FC] column represents the fold change (FC) in the expression level of the indicated gene in *Acsl6*$^{-/-}$ mice in comparison to WT littermates. Genes were sorted by fold change.

Tab "IPA"

Results of Canonical Pathway Analysis. Genes from Tab "DE" were analyzed using the Qiagen Ingenuity Pathway Analysis Software with default parameters. **The ratio** represents the fraction of genes from a given pathway that undergo a change divided by the total number of genes assigned to the same pathway. Activation z-score is a measure of coordinated directional change in the pathway.

Supplementary Data 2. (Excel Microsoft file

The file includes source data displayed in the figures in the main text.
