# Supplementary material for: Acyl-CoA synthetase 6 controls rod photoreceptor function and survival by shaping the phospholipid composition of retinal membranes
Source: Commun Biol. 2024 Aug 21;7:1027. doi: 10.1038/s42003-024-06691-8 (PMC11339274; doi:10.1038/s42003-024-06691-8)
Supplement: Supplementary file 2 — Description of additional supplementary file [file 42003_2024_6691_MOESM2_ESM.pdf]

Description of Additional Supplementary Files

File name: Supplementary Data 1

Description: Gene expression analysis shown in Figure 6 a, b.

File name: Supplementary Data 2

Description: Source data presented in figures of main text.
